# Identification of a New Hesperornithiform from the Cretaceous Niobrara Chalk and Implications for Ecologic Diversity among Early Diving Birds

**DOI:** 10.1371/journal.pone.0141690

**Published:** 2015-11-18

**Authors:** Alyssa Bell, Luis M. Chiappe

**Affiliations:** The Dinosaur Institute, Natural History Museum of Los Angeles County, Los Angeles, California, United States of America; Royal Belgian Institute of Natural Sciences, BELGIUM

## Abstract

The Smoky Hill Member of the Niobrara Chalk in Kansas (USA) has yielded the remains of numerous members of the Hesperornithiformes, toothed diving birds from the late Early to Late Cretaceous. This study presents a new taxon of hesperornithiform from the Smoky Hill Member, *Fumicollis hoffmani*, the holotype of which is among the more complete hesperornithiform skeletons. *Fumicollis* has a unique combination of primitive (e.g. proximal and distal ends of femur not expanded, elongate pre-acetabular ilium, small and pyramidal patella) and derived (e.g. dorsal ridge on metatarsal IV, plantarly-projected curve in the distal shaft of phalanx III:1) hesperornithiform characters, suggesting it was more specialized than small hesperornithiforms like *Baptornis advenus* but not as highly derived as the larger *Hesperornis regalis*. The identification of *Fumicollis* highlights once again the significant diversity of hesperornithiforms that existed in the Late Cretaceous Western Interior Seaway. This diversity points to the existence of a complex ecosystem, perhaps with a high degree of niche partitioning, as indicated by the varying degrees of diving specializations among these birds.

## Introduction

The Cretaceous Hesperornithiformes were one of the first lineages of Mesozoic birds discovered [1]. With their earliest stratigraphic record represented by several taxa (i.e., *Enaliornis barretti*, *E*. *sedgewicki*, and *E*. *seeleyi*) from marine deposits of the Albian of England, the Hesperornithiformes constitute the oldest known major radiation of aquatic birds. The fossilized remains of these highly specialized foot-propelled divers are known from a broad geographic and stratigraphic range, even at low taxonomic levels. One of the earliest recognized genera, *Baptornis*, is known from the Lincoln Limestone (Cenomanian [2]) and Niobrara Chalk (Smoky Hill Member, Coniacian [3]) of Kansas, the Pierre Shale of South Dakota (Campanian), Campanian strata in Sweden [4], and Maastrichtian deposits in Mongolia [5]. Despite this broad distribution, many specimens of *Baptornis* are represented by isolated elements, making taxonomic assignments difficult.

**Fig 1:**
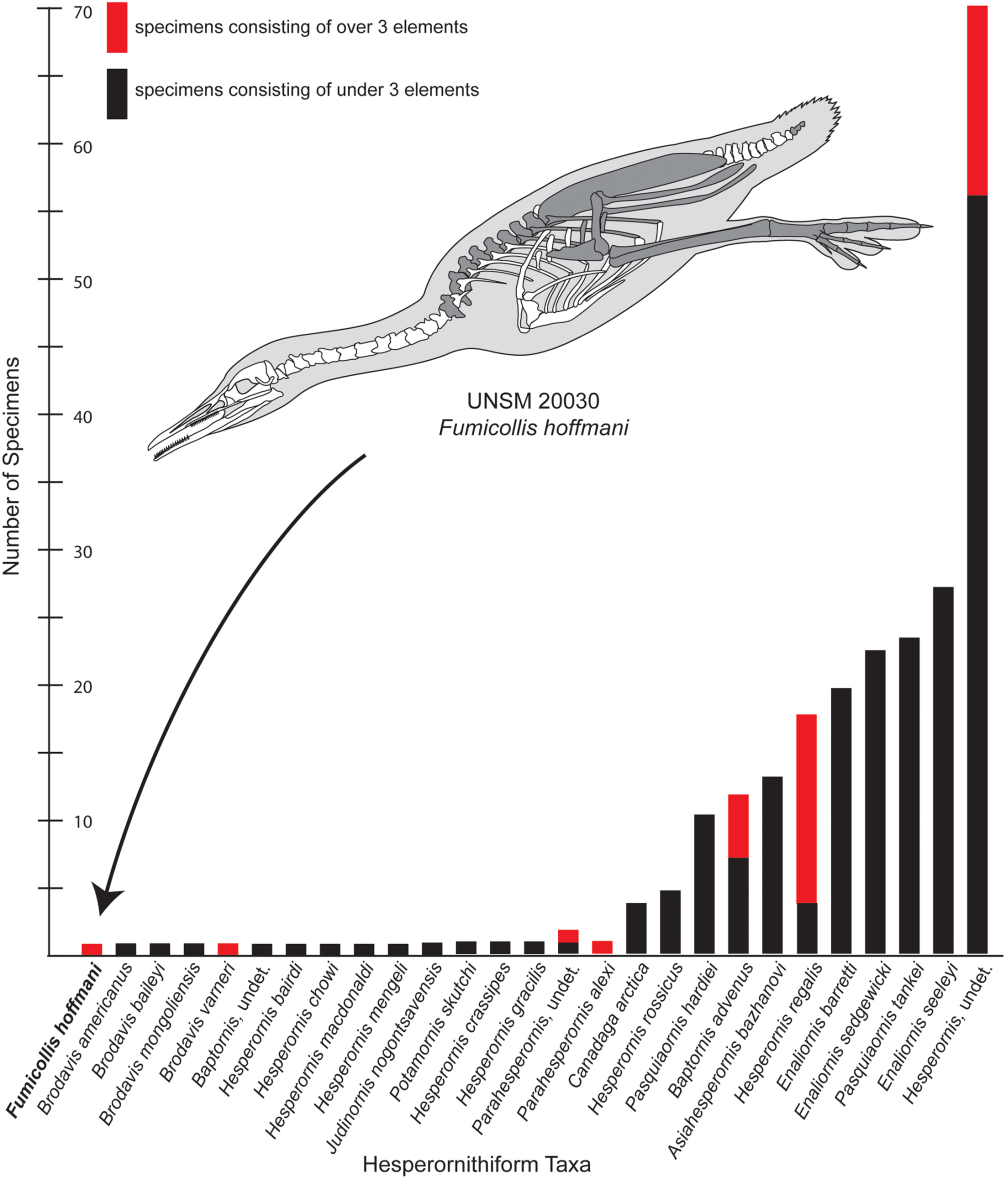
Specimen completeness among reported hesperornithiforms. The number of specimens preserving fewer than three elements (black) and three elements or more (red) are shown for all described hesperornithiform taxa. A schematic reconstruction of UNSM 20030 is shown with the preserved elements shaded dark gray. It should be noted that the exact rib placement is not known and was estimated for the figure. References for hesperornithiform taxa are as follows: *Baptornis advenus* [11, 6]; *Asiahesperornis bazhanovi* [25]; *Brodavis* [26]; *Canadaga arctica* [17, 27]; *Enaliornis* [28]; *Hesperornis crassipes*, *H*. *gracilis* [11]; *H*. *bairdi*, *H*. *chowi*, *H*. *macdonaldi*, *H*. *mengeli* [29]; *H*. *regalis* [11, 30–31]; *H*. *rossicus* [32]; *Judinornis nogontsavensis* [33]; *Parahesperornis alexi* [15]; *Pasquiaornis* spp. [34–36]; *Potamornis skutchi* [37].

*Baptornis advenus* was originally described from an isolated tarsometatarsus (YPM 1476 [3]). While Marsh described this element as “nearly perfect” [3, pg. 80], only the distal half of the element is preserved today. Since Marsh’s work, a few more complete specimens have been assigned to the species on the basis of the morphology of the distal tarsometatarsus, enabling the skeletal description of the species to expand beyond that element [6]. To date, only five specimens of *B*. *advenus* have been reported that preserve more than a single element (although more may be present in museum collections). This dearth of even partially complete specimens is common in hesperornithiform paleontology, where few taxa are known from more than a single element (Fig 1).

**Fig 2:**
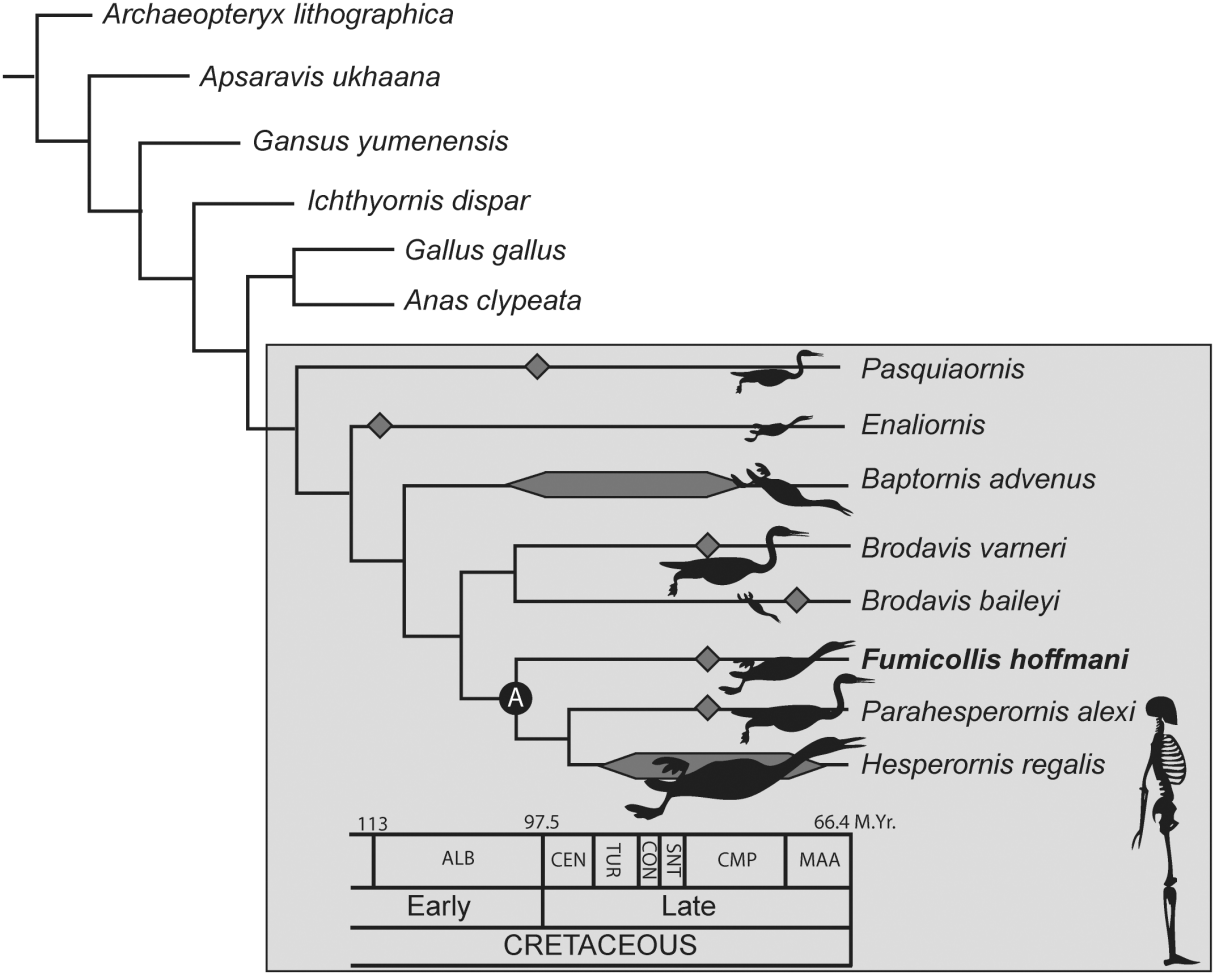
Phylogenetic analysis of Bell and Chiappe (2015) showing the derived placement of *Fumicollis hoffmani* within the Hesperornithiformes (grey box). Time calibration is based on reported ages of known fossils. Body silhouettes are shown to represent approximate size differences among hesperornithiforms, scaled to a 6 foot human (lower right) based on geometric scaling of the tarsometatarsi and femora of known specimens. Node A is united by five unambiguous synapomorphies (see [8] for details). Abbreviations: ALB, Albian; CEN, Cenomanian; CMP, Campanian; CON, Coniacian; MAA, Maastrichtian; SNT, Santonian; TUR, Turonian.

**Fig 3:**
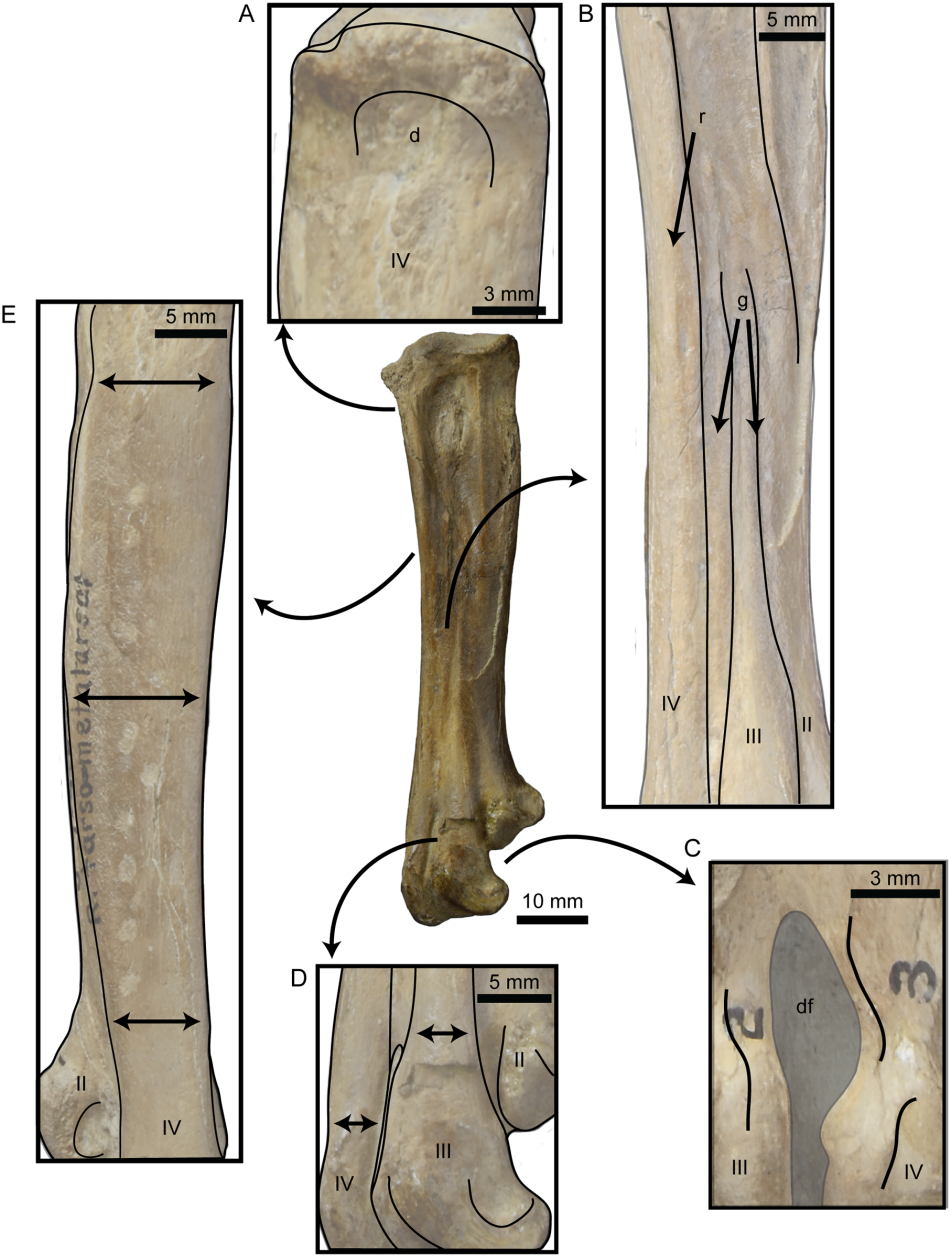
Features of the right tarsometatarsus of UNSM 20030 that were identified by a phylogenetic analysis (see [8]) as uniting *Fumicollis hoffmani*, *Parahesperornis*, and *Hesperornis* in a clade. A, well-developed depression (d) on the lateral face of the proximal end of metatarsal IV; B, prominent grooves (g) separating the metatarsals along the entire length of shaft and ridge (r) that forms the dorsal surface of metatarsal IV; C, the enclosed distal vascular foramen (df) between the trochlea of metatarsals III and IV is tear-drop shaped in plantar view (note the trochleae of the specimen have been labelled incorrectly); D, metatarsals IV, III, and II progressively displaced plantarly (shingled; displacement shown with arrows); and E, shaft of metatarsal IV is widest at midshaft and tapers at both the proximal and distal ends (widths indicated with arrows). Abbreviations: d, depression; df, distal foramen; g, groove; r, ridge; II–IV, metatarsal II–IV.

In 1937 Harold Shepherd and George Sternberg collected a partial post-cranial skeleton of a small hesperornithiform from the Smoky Hill Member of the Niobrara Chalk of Logan County, Kansas (United States). The specimen was obtained by the University of Nebraska State Museum (as UNSM 20030), and was initially referred to *Baptornis advenus* by Martin and Tate [6]. However, recent phylogenetic work by Bell ([7]; see also [8]) discovered that UNSM 20030 conflicts with the holotype of *B*. *advenus* in four morphological characters of the tarsometatarsus and with other specimens of *B*. *advenus* (AMNH 5101, FHSM 6318, FMNH 395, KUVP 2290, KUVP 16112) in an additional 18 characters (see S1 Appendix). These analyses also recovered UNSM 20030 as more closely related to the clade *Parahesperornis* + *Hesperornis* than to *Baptornis* (Fig 2), warranting the erection of a new taxon for UNSM 20030. Fig 3 details the features of the tarsometatarsus of UNSM 20030 that are also found in *Hesperornis* and *Parahesperornis* but not in *Baptornis*.

UNSM 20030 is significant in that it is one of the best-preserved small hesperornithiforms known, with over twenty elements preserved, primarily from the pelvic limb as well as thoracic and posterior cervical vertebrae. As a member of a group of highly derived diving birds where 18 of the 23 named species are known from specimens consisting of fewer than three elements, UNSM 20030 provides the opportunity to better understand the evolution of diving adaptations among the earliest known fully aquatic birds. Furthermore, detailed, well-illustrated descriptive work is rare in hesperornithiform studies, where the seminal anatomical reference remains Marsh’s 1880 monograph, despite the plethora of taxa that have been named in the subsequent 135 years.

### Nonenclatural acts

The electronic edition of this article conforms to the requirements of the amended International Code of Zoological Nomenclature, and hence the new names contained herein are available under that Code from the electronic edition of this article. This published work and the nomenclatural acts it contains have been registered in ZooBank, the online registration system for the ICZN. The ZooBank LSIDs (Life Science Identifiers) can be resolved and the associated information viewed through any standard web browser by appending the LSID to the prefix “http://zoobank.org/”. The LSID for this publication is: urn:lsid:zoobank.org:pub:B831DCE8-1993-4F25-8A41-65038573253A. The electronic edition of this work was published in a journal with an ISSN, and has been archived and is available from the following digital repositories: PubMed Central, LOCKSS [author to insert any additional repositories].

## Systematic Paleontology

Aves Linnaeus 1758

Ornithuromorpha Chiappe 2002

Ornithurae Haeckel 1866

Hesperornithiformes Fürbringer 1888

*Fumicollis hoffmani* gen. et sp. nov.

urn:lsid:zoobank.org:act:81992545-5F0F-4ED4-BEE4-9825ACCDE769

urn:lsid:zoobank.org:act:D8E670C5-1E02-4308-B34B-B3E1F43AABF3

### Etymology

The genus name *Fumicollis* is from the Latin fumi (smoke) and collis (hill), in reference to the Smoky Hill Member of the Niobrara Chalk in which the specimen was discovered. The species name *hoffmani* is in recognition of Karen and Jim Hoffman, whose generous support has greatly enhanced the programs of the Natural History Museum of Los Angeles County, including research at the Dinosaur Institute.

### Holotype

The holotype and only known specimen preserves eight presacral vertebrae; multiple rib fragments; the right half of the pelvis including a nearly complete ilium, ischium, and pubis; portions of the left half of the pelvis, including the acetabulum and a fragment of either the ischium or pubis; a portion of the pygostyle preserving three distal-most fused centra; the nearly complete left hindlimb (femur, patella, fibula, tibiotarsus, tarsometatarsus, and four pedal phalanges); the partial right tibiotarsus; and two right pedal phalanges.

### Occurrence

The Smoky Hill Member of the Niobrara Chalk (upper Coniacian—lower Campanian [9]), five miles northeast of Elkader in Logan County, Kansas.

### Diagnosis

UNSM 20030 is unique from other hesperornithiform birds in possessing a suite of features that represent a combination of traits known from either *Baptornis* or *Hesperornis* and *Parahesperornis*. The following unique combination of features is diagnostic of *Fumicollis hoffmani*: presacral vertebrae with expanded ventral processes; elongate pelvis with reduced acetabulum (acetabulum width: pelvis length is approximately 0.096); femur with expanded lateral condyle (transverse extent of condyle over 75% of midshaft width) and moderately expanded trochanter (transverse extent of trochanter nearly half of midshaft width); tibiotarsus with triangular cnemial expansion and medial cnemial crest extended to midshaft; patella pyramidal with flattened caudal face and perforated for ambiens tendon; fibula with caudally-expanded proximal end and slightly depressed, saddle-shaped articular surface; tarsometatarsus with stacked or shingled metatarsals; distinct dorsal ridge of the tarsometatarsus formed by the entire length of metatarsal IV; tarsometatarsus with reduced and plantarly displaced trochlea II; enlarged medial trochlear ridge of metatarsal IV; phalanx III:1 with narrow medio-lateral width and greatly expanded, curved medial face of the distal end.

## Description

The majority of anatomical nomenclature used in this study follows Baumel and Witmer [10], unless otherwise noted.

### Vertebrae

**Fig 4:**
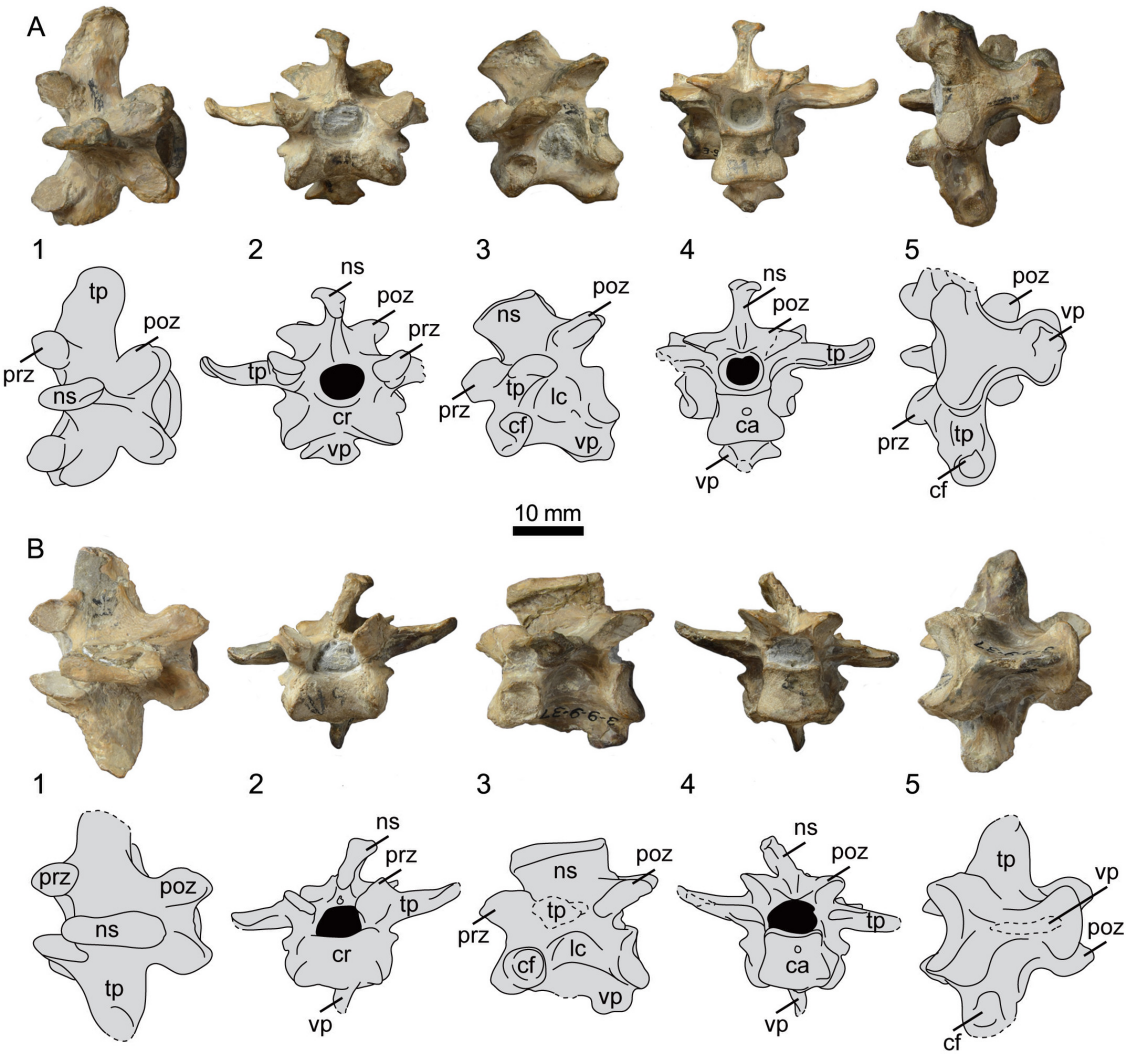
Photographs and interpretative drawings of presacral vertebrae 16 (A) and 17 (B) of UNSM 20030 in dorsal (1), cranial (2), lateral (3), caudal (4), and ventral (5) views. Abbreviations: ca, caudal articular surface; cf, costal fovea; cr, cranial articular surface; lc, lateral concavity; ns, neural spine; poz, postzygapophysis; prz, prezygapophysis; tp, transverse process; vp, ventral process.

UNSM 20030 preserves eight presacral vertebrae, most likely corresponding in position to the sixteenth to twenty-third presacral vertebrae, as determined through comparison to the well-known axial skeleton of *Hesperornis regalis* ([11]; in birds vertebral numbering starts with the atlas, which is counted as the first presacral—see [10]). Select measurements of the vertebrae of UNSM 20030 are found in Table 1. The positions of the vertebra of UNSM 20030 were identified primarily through comparison with Marsh’s sequence of the vertebrae of *Hesperornis regalis* (YPM 1207 and YPM PU 18589)[11], but also through comparison with the extremely well-preserved vertebral column of *Parahesperornis alexi* (KUVP 2287 and KUVP 24090) and the more fragmentary vertebral column of *Baptornis advenus* (AMNH 5101, KUVP 2280b, KUVP 2290, KUVP 16112). In hesperornithiforms, a number of vertebrae are characterized by anatomically unique ventral processes that make identifying specific placement within the vertebral column possible. Furthermore, while the vertebrae of UNSM 20030 were not preserved in articulation, they articulate fairly well in the order in which they have been identified. The cranial-most preserved vertebra of UNSM 20030 is the sixteenth presacral vertebra. This vertebra is well-preserved, lacking only the left transverse process (Fig 4a). This vertebra is identified as the sixteenth presacral by the presence of a short, robust ventral process with a spade-like appearance, much like that of *H*. *regalis* (see YPM 1206), on the cranial end of the ventral surface (best seen in caudal and lateral views, Fig 4a). In cranial view, the vertebra appears short and squat. The cranial zygapohyses are widely spread with the flat surfaces angled inward toward the midline of the centrum. In caudal view, the caudal articular surface is much narrower than the cranial, giving the vertebra a more elongate appearance than in cranial view. The caudal zygapophyses are much more flattened than in *H*. *regalis*. A thickened ridge along the dorsal-most margin of the neural spine is most obvious in caudal view. The neural spine is completely preserved, however the shape may be slightly deformed. The dorsal-most edge of the neural spine is thickened compared to the rest of the spine, resulting in a ridge running along the upper edge. This ridge is also seen in *H*. *regalis* and *P*. *alexi*, however the corresponding elements are unpreserved in *B*. *advenus*. The lateral concavities of the centrum appear to be circular, however the degree of excavation is obscured by sediment. A round costal fovea is present on the ventral-most margin of the cranial end of the centrum, below the transverse process. In ventral view the base of the centrum is highly asymmetricalal, with a much broader cranial end and a slightly caudally-displaced waist. The preserved transverse process is wide and blunt, curving caudally in dorsal view. In cranial or caudal view, the tips of the transverse processes curve upward, however to a much lesser degree than that observed in *H*. *regalis*.

**Table 1:** Selected measurements of the presacral vertebrae of UNSM 20030. All measurements are in millimeters. Asterisk indicates approximate measurement.

Vertebrae	Centrum Length	Width of Cranial Articular Surface
16	16.87	16.97
17	15.34	12.85
18	16.72	13.08
19	16.51	12.28
20	15.89	14.85
21	16.7	12.36*
23	13.99	18.72

The seventeenth presacral vertebrae of UNSM 20030 is slightly deformed, with the dorsal half of the vertebra more poorly preserved than the ventral half (Fig 4b). The ventral process is broken at an angle near the base of the centrum, with the caudal end preserving a small portion of the edge of the process. The entire dorsal process is preserved, however it is tilted laterally. A thickened ridge along the dorsal-most edge is present. Both transverse processes are partially preserved, lacking the lateral-most end. In ventral view the surface of the centrum is more symmetrical than that of the sixteenth vertebra, with less discrepancy in the widths of the wider cranial end and the narrower caudal end. The middle region narrows into a waist, more so than in the sixteenth vertebra. The lateral excavations of the centrum are circular and located centrally, however the degree of excavation is not constant, with the right side more deeply excavated than the left. A large, circular costal fovea is present on the cranio-ventral edge of the lateral surface of the centrum.

**Fig 5:**
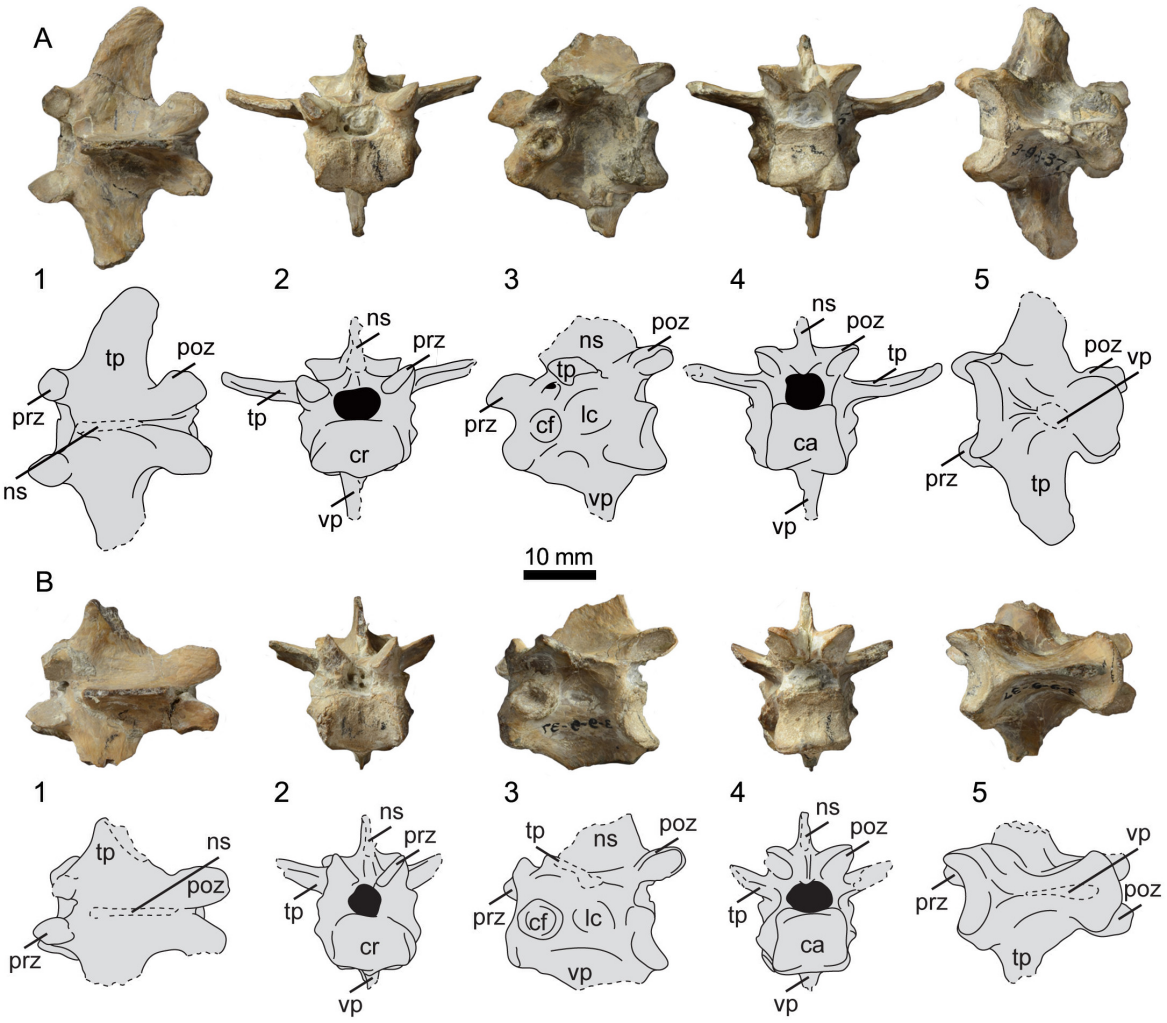
Photographs and interpretative drawings of presacral vertebrae 18 (A) and 19 (B) of UNSM 20030 in dorsal (1), cranial (2), lateral (3), caudal (4), and ventral (5) views. Abbreviations: ca, caudal articular surface; cf, costal fovea; cr, cranial articular surface; lc, lateral concavity; ns, neural spine; poz, postzygapophysis; prz, prezygapophysis; tp, transverse process; vp, ventral process.

The eighteenth vertebra of UNSM 20030 preserves a portion of the dorsal process, a small piece of the ventral process, the entire right transverse process, and a portion of the left (Fig 5a). The dorsal process is positioned caudally on the dorsal surface of the centrum, as in other hesperornithiforms. It appears to be less restricted cranio-caudally than in *P*. *alexi* or *H*. *regalis*, and more closely resembles *B*. *advenus* (AMNH 5101) in this feature. In dorsal view, the caudal zygapophyses are rounded and angle laterally, as in *H*. *regalis* and unlike *B*. *advenus*, where they are narrower and more closely set to the midline of the centrum. The lateral concavities are shallowly excavated on either side of the centrum and a small, circular costal fovea is present along the midline of the centrum at the cranial end. This fovea is more dorsally located than in the preceding vertebrae of UNSM 20030. In ventral view the surface of the centrum is similar to that of the seventeenth vertebra. The fully preserved transverse process is short and curved caudally in dorsal view and angles slightly dorsally in cranial or caudal view. Like in the sixteenth vertebrae, the transverse processes of UNSM 20030 are not turned dorsally at the tips to the same degree as that observed in *H*. *regalis*.

**Fig 6:**
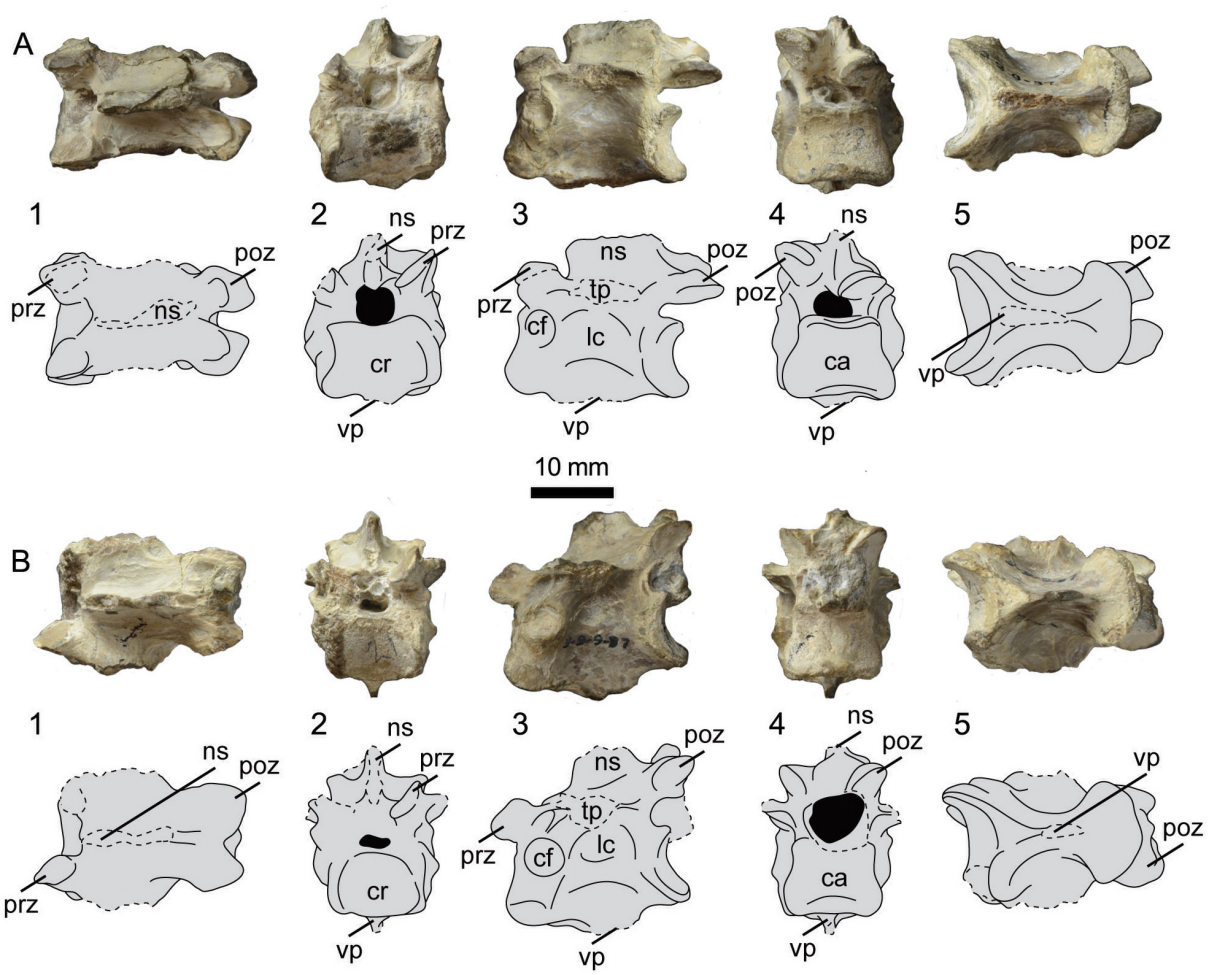
Photographs and interpretative drawings of presacral vertebrae 20 (A) and 21 (B) of UNSM 20030 in dorsal (1), cranial (2), lateral (3), caudal (4), and ventral (5) views. Abbreviations: ca, caudal articular surface; cf, costal fovea; cr, cranial articular surface; lc, lateral concavity; ns, neural spine; poz, postzygapophysis; prz, prezygapophysis; tp, transverse process; vp, ventral process.

The nineteenth, twentieth, and twenty-first vertebrae of UNSM 20030 are poorly preserved and difficult to differentiate from each other (Figs 5b and 6). None of these vertebrae preserve any distinctive features that make specific identification certain. All preserve evidence of a caudally-offset dorsal process, ventral surface of the centrum with an hourglass shape and ventral processes, and closely-spaced cranial and caudal zygapophyses. All of these features are consistent with the thoracic vertebrae of hesperornithiform birds. In ventral view, the edge of the cranial articular surfaces appears to be narrow, as in *P*. *alexi*, instead of the broader opening seen in *B*. *advenus* and *H*. *regalis*, however to what degree this is influenced by taphonomy is unclear.

**Fig 7:**
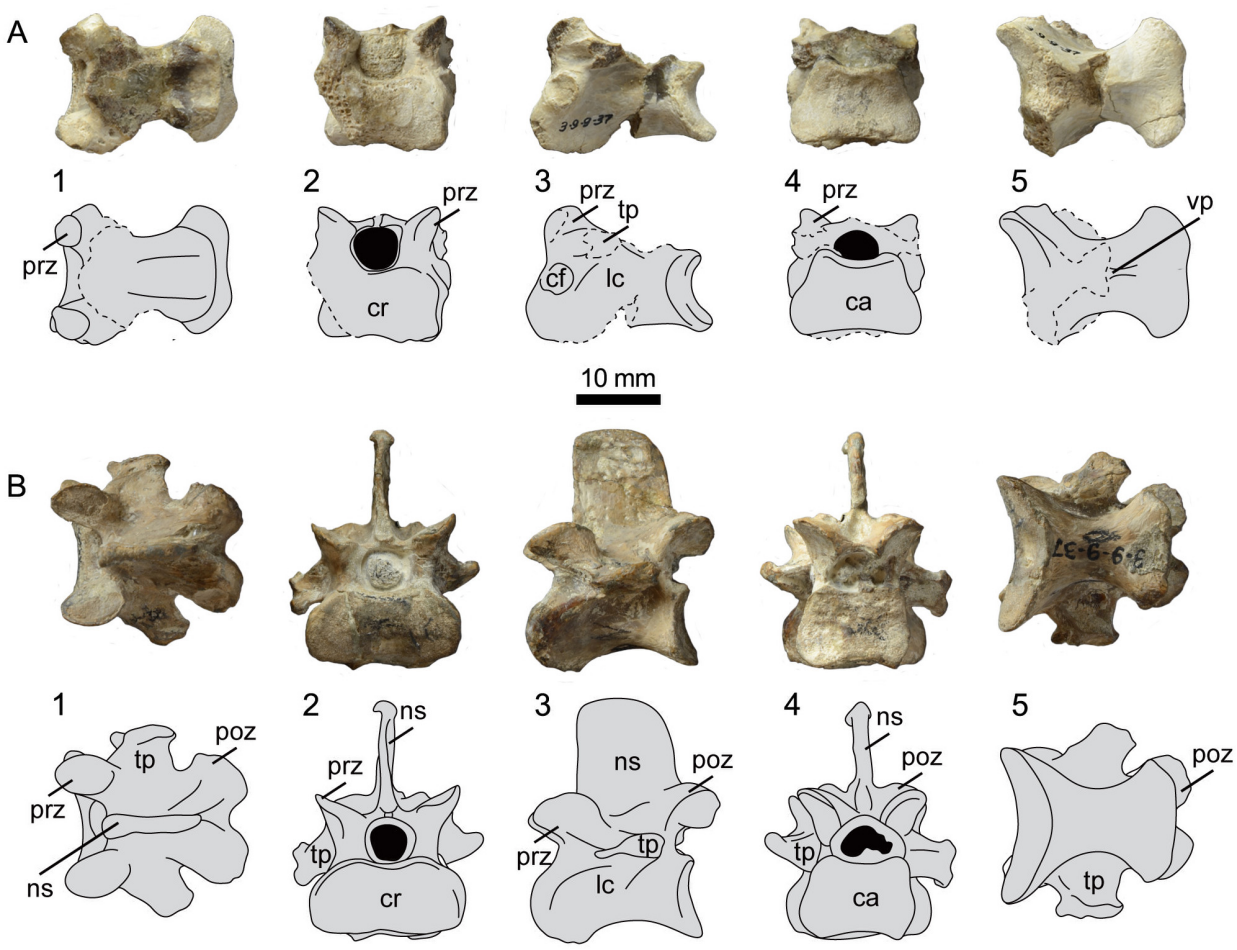
Photographs and interpretative drawings of presacral vertebrae 22 (A) and 23 (B) of UNSM 20030 in dorsal (1), cranial (2), lateral (3), caudal (4), and ventral (5) views. Abbreviations: ca, caudal articular surface; cf, costal fovea; cr, cranial articular surface; lc, lateral concavity; ns, neural spine; poz, postzygapophysis; prz, prezygapophysis; tp, transverse process; vp, ventral process.

The twenty-second vertebra is poorly preserved, lacking the dorsal half of the centrum, including the transverse processes (Fig 7a). This vertebra is only identified as the twenty-second by the shortening of the broken surface left by the ventral process on the ventral surface of the centrum, as compared to other vertebrae in the series. In hesperornithiform birds, the ventral process becomes increasingly cranio-caudally restricted along the ventral surface in the most caudal thoracic vertebrae, with the twenty-second having only a small portion of the cranial surface occupied by the ventral process, as seen in this vertebrae from UNSM 20030. The dorsal surface of the twenty-second vertebra forms a symmetrical hour-glass shape, with a waist present near the midline of the centrum and the cranial and caudal articular surfaces nearly equal in width, as in other hesperornithiforms.

The twenty-third and final vertebra before the synsacrum is one of the better-preserved thoracic vertebrae of UNSM 20030, with the complete dorsal process and centrum, as well as portions of both transverse processes (Fig 7b). Like other hesperornithiforms, the ventral surface of the centrum is broad and smooth, with no ventral process. The ventral surface is hourglass-shaped, with the waist located near the midline of the centrum and the cranial end more expanded than the caudal. The neural spine is much taller than in the other preserved vertebra, also consistent with hesperornithiforms, where the relative height of the neural spine increases caudally along the vertebral column. However, the neural spine is proportionally shorter than that of *H*. *regalis* (YPM 1207), where it extends to a height that is greater than the dorso-ventral height of the centrum. In UNSM 20030, the neural spine is similar to the dorso-ventral height of the centrum. The neural spine of the twenty-third vertebrae is not preserved in any specimen of *B*. *advenus*. The thickened ridge at the top of the neural spine is less robust than that seen in the more cranial vertebrae of UNSM 20030, a pattern also observed in *H*. *regalis*.

### Pelvis

**Fig 8:**
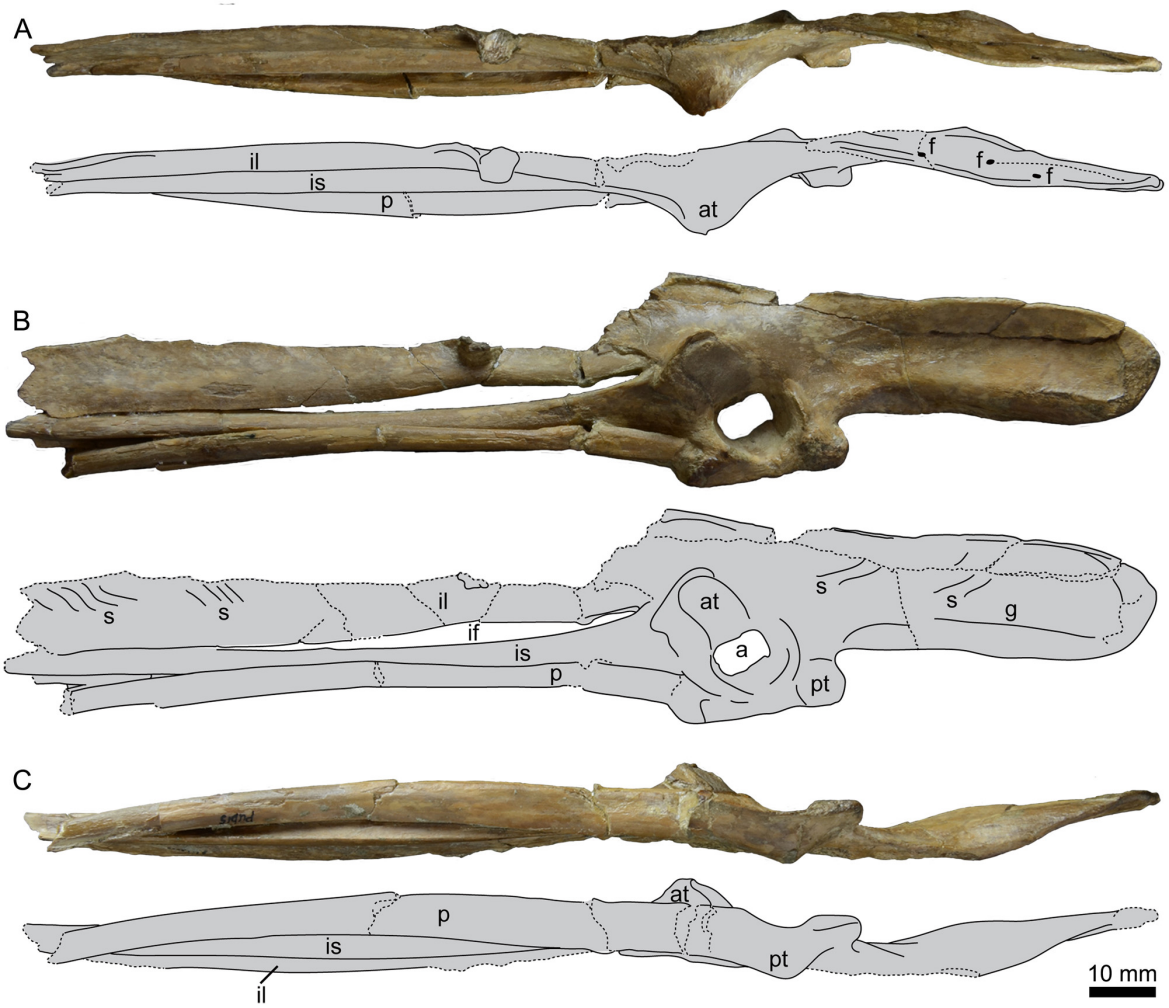
Photographs and interpretative drawings of the right ilium, ischium, and pubis of UNSM 20030 in dorsal (A), lateral (B), and ventral (C) views. Abbreviations: a, acetabulum; at, antitrochanter; f, foramen; g, groove; if, Ilioischiadic fenestra; il, ilium; is, ischium; p, pubis; pt, preacetabular tubercle; s, sinusoidal grooves.

**Fig 9:**
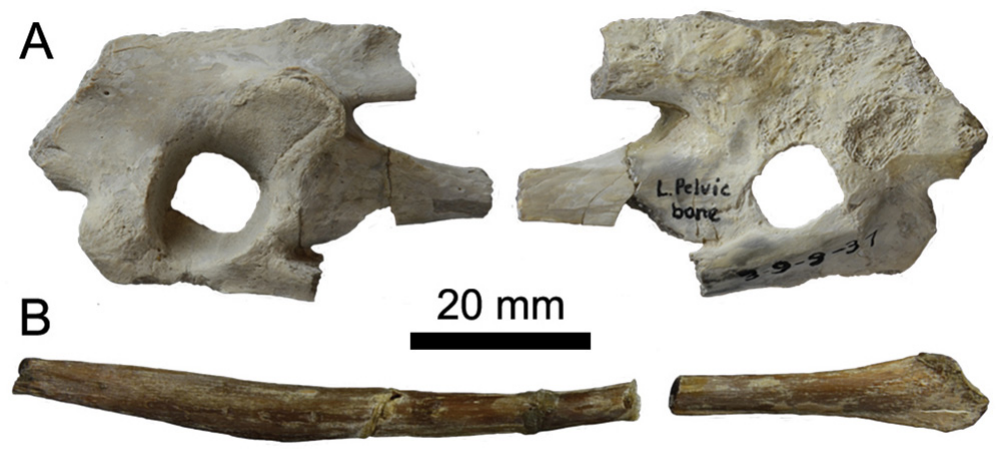
Photographs of the left acetabular region (A) in lateral (left) and medial (right) views and portions of the left ischium or pubis (B) of UNSM 20030 in lateral view.

The right side of the pelvis is fairly complete, lacking the caudal-most end of the ischium and pubis and a portion of the body of the post-acetabular ilium (Fig 8), while the left side of the pelvis is represented only by the acetabulum and an element that may be a portion of either the pubis or ischium (Fig 9). The pre-acetabular ilium is elongate, comprising 36% of the total preserved length. As most of the original length appears to be preserved, this percentage would probably be only slightly reduced in the complete ilium. The holotype of *P*. *alexi* (KUVP 2287) is the only hesperornithiform to preserve a complete ilium, with a pre-acetabular ilium that is 31% of the total length. The most complete pelvis of *H*. *regalis* (YPM 1476) is missing the distal-most extent of the ilium, similar to the case in UNSM 20030, and in that specimen the pre-acetabular ilium comprises 30% of the total ilium length. The lateral face of the pre-acetabular ilium has a longitudinal depression along the cranial portion, which is more distinct than in *H*. *regalis* (YPM 1207 and YPM 1476). Unlike *H*. *regalis*, the lateral surface of the pre-acetabular ilium is relatively smooth, with the rough, wavy appearance of *Hesperornis* only faintly visible. A prominent, rounded pre-acetabular tubercle is present at the ventral margin of the pre-acetabular ilium. While not preserved in most hesperornithiform specimens, the pre-acetabular tubercle of *P*. *alexi* forms an elongate flange while that of *H*. *regalis* appears to be more pointed, however the size cannot be determined due to breakage. The acetabulum of UNSM 20030 is fairly small (9% of preserved length) with an irregular outline. This size is similar to that observed in other hesperornithiforms (7% in *P*. *alexi* and 10% in *H*. *regalis*), however the incomplete preservation of some of these specimens makes these values somewhat questionable. The walls of the acetabulum slope very steeply, being nearly vertical, with a pronounced lip on the cranial edge. It is impossible to say if the acetabulum was perforated or not, as the interior is broken away with no natural edges preserved. The antitrochanter is positioned caudo-dorsally from the acetabulum (centered on 10 o’clock from the center of the acetabulum) and merges smoothly into the wall of the acetabulum. The overall shape of the antitrochanter is boxy, forming a smooth slope from the ventral margin down to the edge of the acetabulum. The cranial portion of the surface of the antitrochanter appears to have a raised surface distinct from the remainder of the face of the antitrochanter. The dorso-ventrally highest portion of the antitrochanter ios over 57% of the total dorso-ventral height of the pelvis at the acetabulum, the same as is observed in *H*. *regalis* and *P*. *alexi*. In dorsal view, the antitrochanter of UNSM 20030 protrudes slightly from the wall of the ilium, very similar to the degree of protrusion seen in *P*. *alexi*. This is slightly less than that of *H*. *regalis* and markedly less than the protrusion of the antitrochanter of *B*. *advenus* (AMNH 5101).

The post-acetabular ilium is not well-preserved, with a large section of the dorsal half absent. The surface is very smooth and gently bulges into a rounded ventral-most surface with some light striations visible near the caudal end. This differs from *H*. *regalis*, where the ventral margin of the ilium is flattened, with a rougher surface. The striations are more visible on the dorsal half of the face of the post-acetabular ilium in *H*. *regalis*, and do not continue all the way to the ventral margin. The appearance of the post-acetabular ilium in UNSM 20030 is most similar to that of *P*. *alexi*, where a similar rounded margin is observed.

The ischium is fused to the ilium caudal to the antitrochanter and acetabulum. The ischium narrows abruptly from the cranial end, and then maintains an even width to the broken caudal-most end. The surface of the ischium of UNSM 20030 appears to be more rounded than in *P*. *alexi*, however to what extent this is due to crushing in *P*. *alexi* is unclear. The pubis is fused to the dorsal acetabulum below the ischium, forming a distinct bulge in the margin of the pelvis caudo-dorsally to the acetabulum and a flattened edge running along the dorsal margin of the pelvis from the pubis, below the acetabulum, to the pre-acetabular tubercle. The shaft of the pubis forms a slight arch along its length and also has a rounded surface.

### Pygostyle

**Fig 10:**
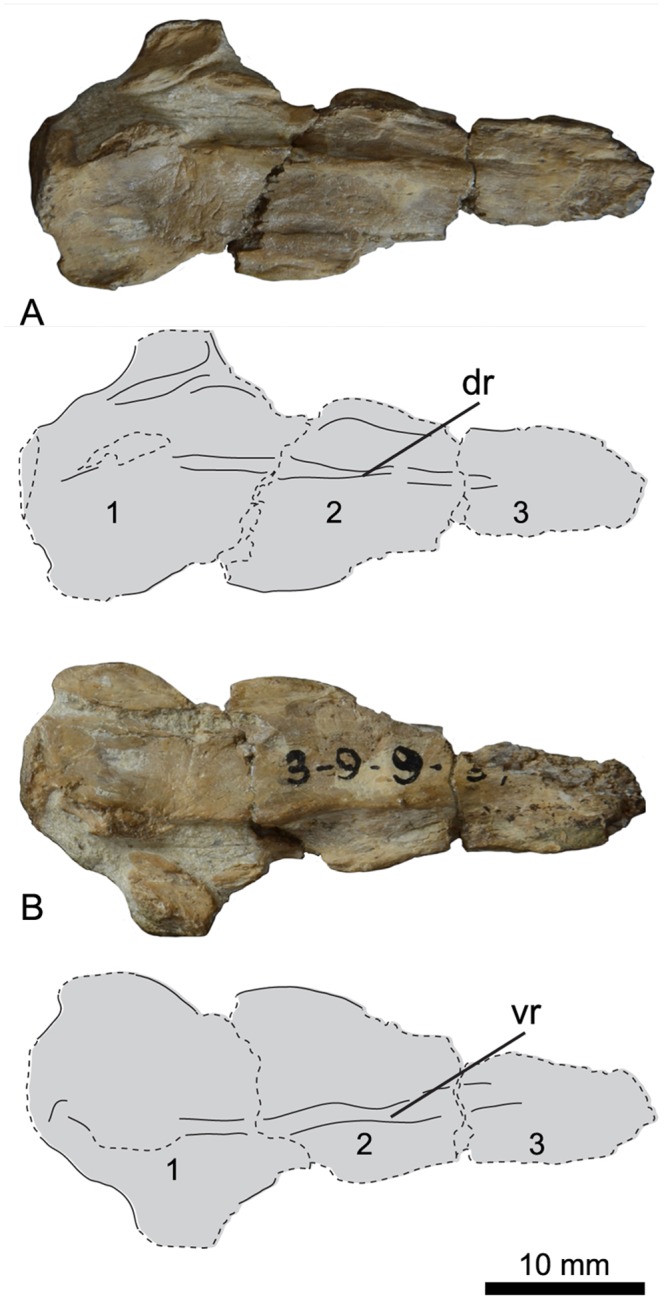
Photographs and interpretative line drawings of the pygostyle of UNSM 20030 in dorsal (A) and ventral (B) views. Centra are numbered. Abbreviations: dr, dorsal ridge; vr, ventral ridge.

A poorly preserved pygostyle with three fused centra is present in UNSM 20030 (Fig 10). Both ends of the pygostyle are broken, and so it is possible that additional centra were included on either end. Very little of the element is preserved beyond the centra, so comparisons to other hesperornithiform pygostyles are difficult. The holotype of *H*. *regalis* (YPM 1200) preserves a number of free caudal vertebrae and a pygostyle with two fused centra and broad transverse processes. A specimen assigned to *P*. *alexi* (KUVP 24090) preserves a complete pygostyle, consisting of three to four fused centra and transverse processes that are somewhat more slender than those of *H*. *regalis*. No pygostyle is known for *B*. *advenus*.

### Femur

**Fig 11:**
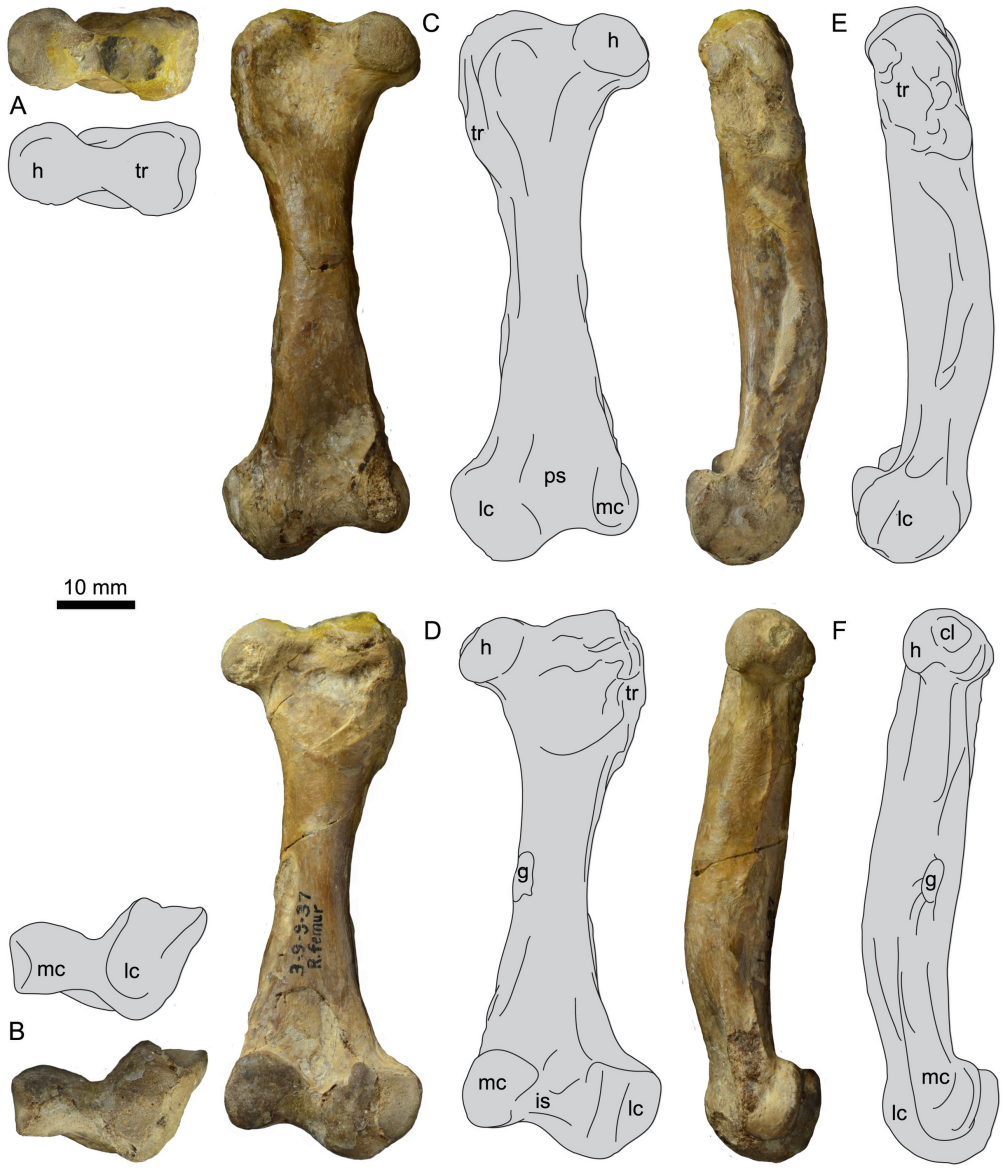
Photographs and interpretative line drawings of the right femur of UNSM 20030 in proximal (A), distal (B), cranial (C), caudal (D), lateral (E), and medial (F) views. Abbreviations: cl, fovea for capital ligament; g, gastrocnemial muscle scar; h, head; is, intercondylar sulcus; lc, lateral condyle; mc, medial condyle; ps, patellar sulcus; tr, trochanter.

**Fig 12:**
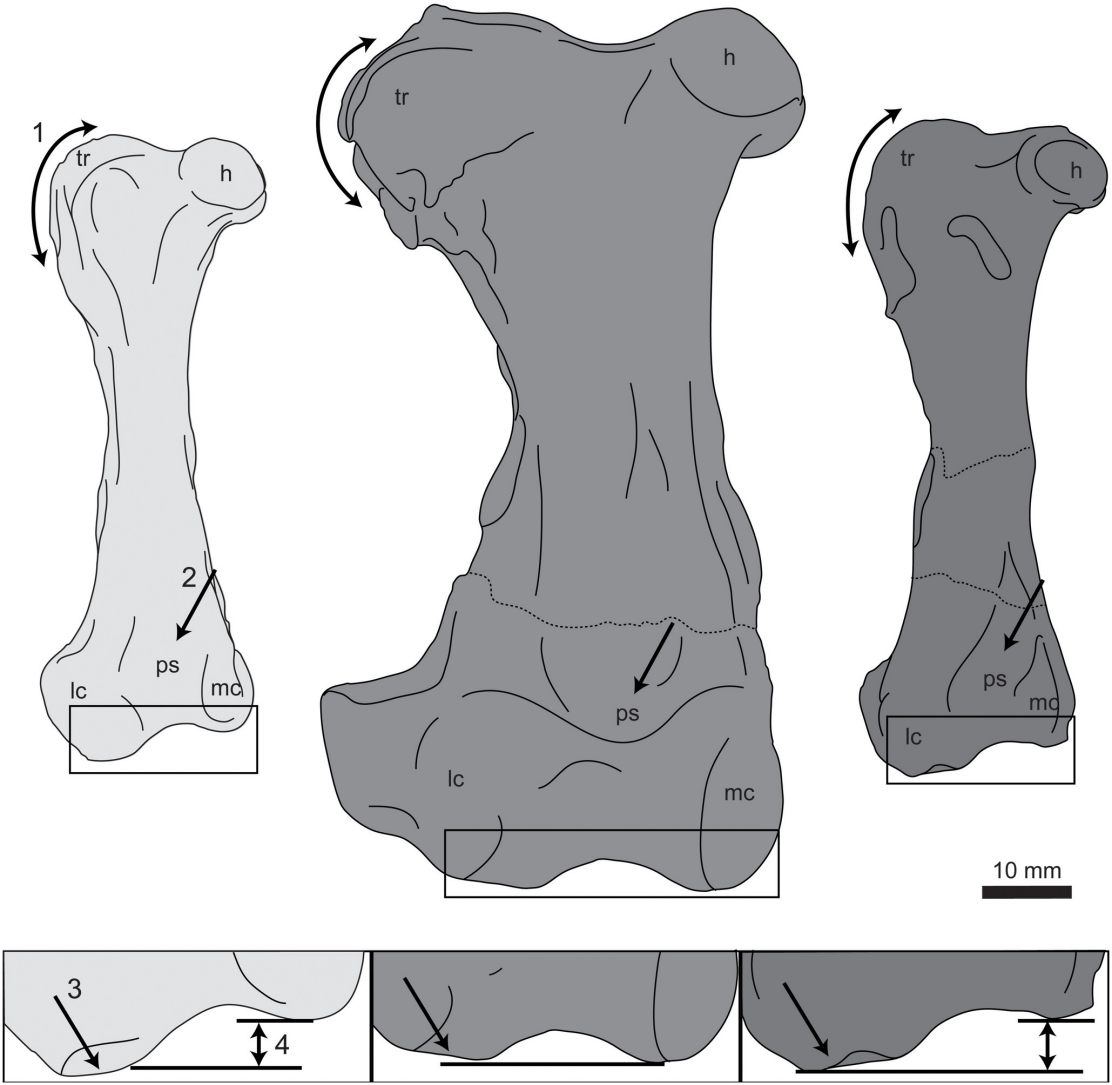
Comparison of the femora of hesperornithiforms in cranial view (left-to-right: *Fumicollis hoffmani* UNSM 20030; *Hesperornis regalis* YPM 1200; *Baptornis advenus* AMNH 5101). 1, arrow indicates the profile of the trochanter, which is rounded in *F*. *hoffmani* and *B*. *advenus* but more angled in *H*. *regalis*. 2, arrow indicates the patellar sulcus, which is pocketed in *H*. *regalis* but not in *F*. *hoffmani* and *B*. *advenus*. Insets: 3, arrow indicates the flattened surface of the lateral condyle; 4, arrow highlights the difference in distal extents of the medial and lateral condyles. In *F*. *hoffmani* and *B*. *advenus* the lateral condyle extends further distally, while in *H*. *regalis* the condyles have a similar extent, with the medial very slightly further than the distal. Insets are shown scaled to be of similar size to *F*. *hoffmani*. Abbreviations: h, head; lc, lateral condyle; mc, medial condyle; ps, patellar sulcus; tr, trochanter.

UNSM 20030 preserves the complete right femur (Fig 11), measurements of which are found in Table 2. The femur bears a number of features found in hesperornithiform birds, such as a robust trochanter, expanded lateral condyle, and rugose muscle scars. The head of the femur is round and separated from the proximal end by a distinct neck. The medial face of the head is flattened and directed proximo-medially, and bears a slight depression for the attachment of the capital ligament. The head is separated from the trochanter by a constriction of the proximal edge of the femur that is visible in cranial or caudal view as well as in proximal view. In proximal view, the head and trochanter are of similar cranio-caudal widths and both appear very rounded, as in *B*. *advenus*. In specimens assigned to *Hesperornis*, this feature is highly variable, with the trochanter appearing highly ovate in the holotype of *H*. *regalis* (YPM 1200; see also *H*. *regalis* YPM 1207, *H*. sp. FMNH 321) but more circular in other specimens (e.g. *H*. *gracilis* YPM 1679, *H*. *regalis* YPM 1476, *H*. sp. AMNH 2181, and *H*. sp. FMNH 321). In cranial view the trochanter forms a thick, smooth ridge along the proximal and lateral margins of the femur, while in caudal view the trochanter is highly rugose (Fig 12a). This is similar to the appearance of the trochanter of *B*. *advenus* and different from that of *H*. *regalis* and *P*. *alexi*, where the trochanter is highly rugose on both the cranial and caudal faces and forms a sharp angle on the lateral-most margin of the trochanter in cranial or caudal view. In lateral view the trochanter is highly flattened, with a number of muscle scars crossing the surface for the attachments of the *m*. *iliotrochantericus caudalis* and *cranialis* as well as the *m*. *obturatorius lateralis* and *mediali*s [12]. The trochanter extends roughly one-quarter of the way down the lateral shaft.

**Table 2:** Select measurements of the femur of UNSM 20030, in millimeters.

Length	Mid-shaft Diameter	Proximal Medio-lateral Width	Distal Medio-lateral Width	Width of Fibular Condyle
71.8	11.34	26.37	28.45	9.24

**Fig 13:**
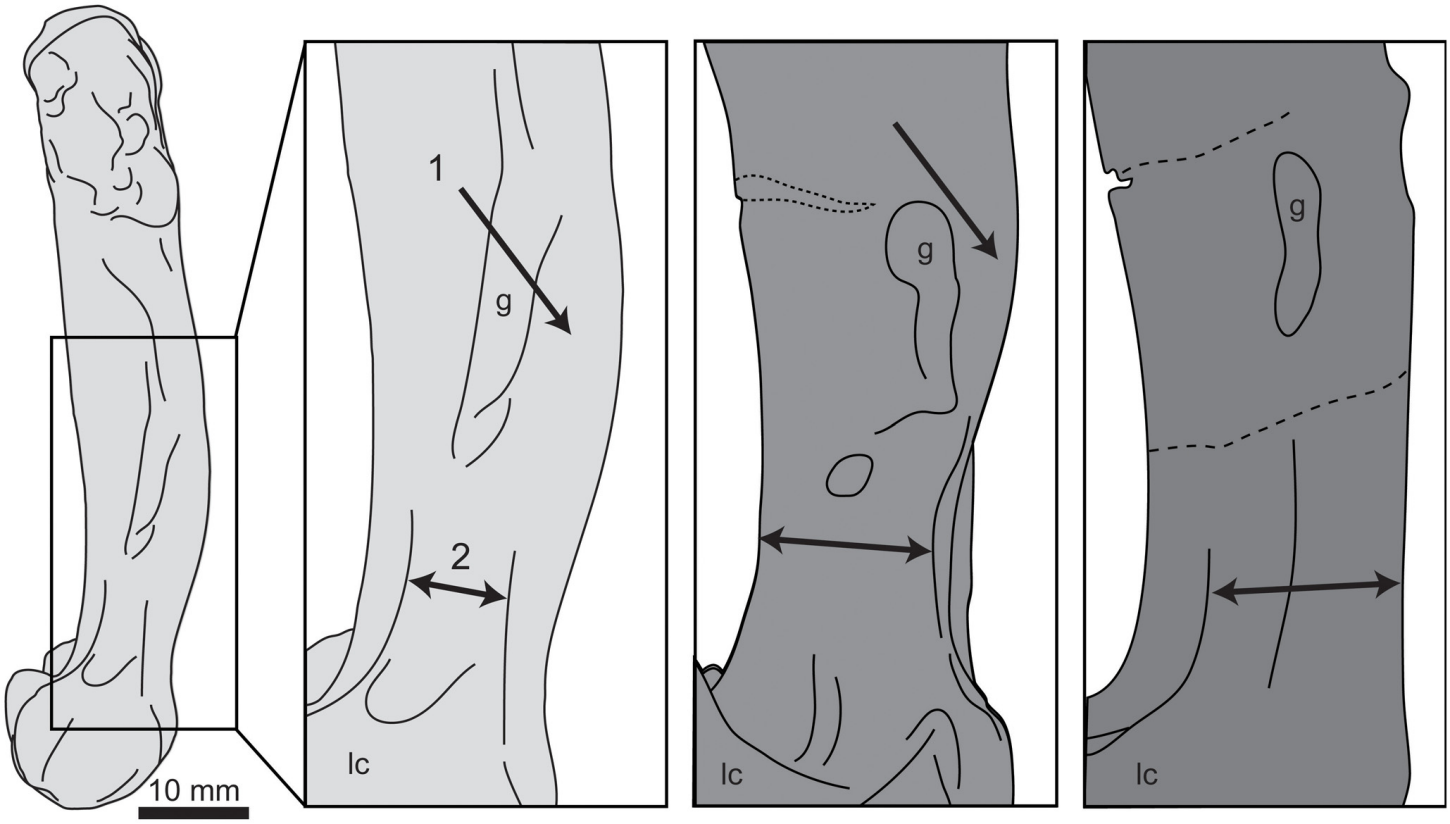
Comparison of the femora of hesperornithiforms in lateral view (left-to-right: *Fumicollis hoffmani* UNSM 20030; *Hesperornis regalis* YPM 1200; *Baptornis advenus* AMNH 5101). 1, arrow indicates the expanded bulge to the cranial surface of the distal shaft seen in *F*. *hoffmani* and *H*. *regalis* but not in *B*. *advenus*. 2, arrow indicates the narrowing of the distal lateral shaft into a neck proximal to the lateral condyle. The degree of narrowing is most extreme in *F*. *hoffmani*, present to a lesser degree in *H*. *regalis*, and nearly absent *B*. *advenus*. Insets are shown scaled to be of similar size to *F*. *hoffmani*. Abbreviations: g, scar for the gastrocnemial muscle; lc, lateral condyle.

In cranial and caudal view the shaft of the femur narrows evenly to a waist near midshaft. The lateral margin of the shaft is more highly indented or curved than the medial, as in other hesperornithiforms. In medial and lateral views the shaft is caudally concave, curving out cranially with a noticeable bulge toward the distal end of the shaft. This shape is also observed in *H*. *regalis* and *P*. *alexi*, while in *B*. *advenus* the shaft is nearly straight in side view (Fig 13a). A prominent muscle scar, possibly for the attachment of the lateral gastrocnemial muscle, is present on the distal-lateral shaft, as in other hesperornithiforms. The distal end of the femur of UNSM 20030 features a reduced medial condyle and an expanded lateral condyle, as is common in all hesperornithiforms. The cranial surface of the medial condyle of UNSM 20030 is somewhat eroded. The caudal surface is slightly wedge-shaped, with the proximal end wider than the distal end, as in most hesperornithiforms, and set at an angle to the shaft, as in *P*. *alexi* and *H*. *regalis*. In medial view the medial condyle is very narrow with a lip extending around the caudal margin of the condyle. The medial shaft of the femur narrows sharply to a thin neck just proximal to the condyle. This narrowing into a neck is more pronounced than in other hesperornithiforms. In cranial view, a slightly indented patellar sulcus is present and merges smoothly into the distal end of the femur. This is also observed in *B*. *advenus* and very different from the distinct patellar sulcus separated from the distal end by a ridge in *H*. *regalis* and *P*. *alexi* (Fig 12b). The lateral condyle is smooth and rounded in cranial view, unlike the oval-shape observed in *H*. *regalis*. The lateral condyle extends much further distally than the medial, as in *B*. *advenus*, while in *H*. *regalis* and *P*. *alexi* the condyles extend a similar distance distally. In caudal view the lateral condyle is angled slightly laterally, with the medial and lateral margins of the caudal face defined by sharp ridges and a slightly depressed center. The distal face of the lateral condyle bears a large bulge with a slightly flattened distal end, similar to that seen in *H*. *regalis* and *P*. *alexi*, which is not observed in *B*. *advenus* (Fig 12c). In lateral view the shaft of the femur narrows to a slight neck just above the lateral condyle, a feature also seen in *H*. *regalis* and *P*. *alexi* but not in *B*. *advenus* (Fig 13b).

### Patella

**Fig 14:**
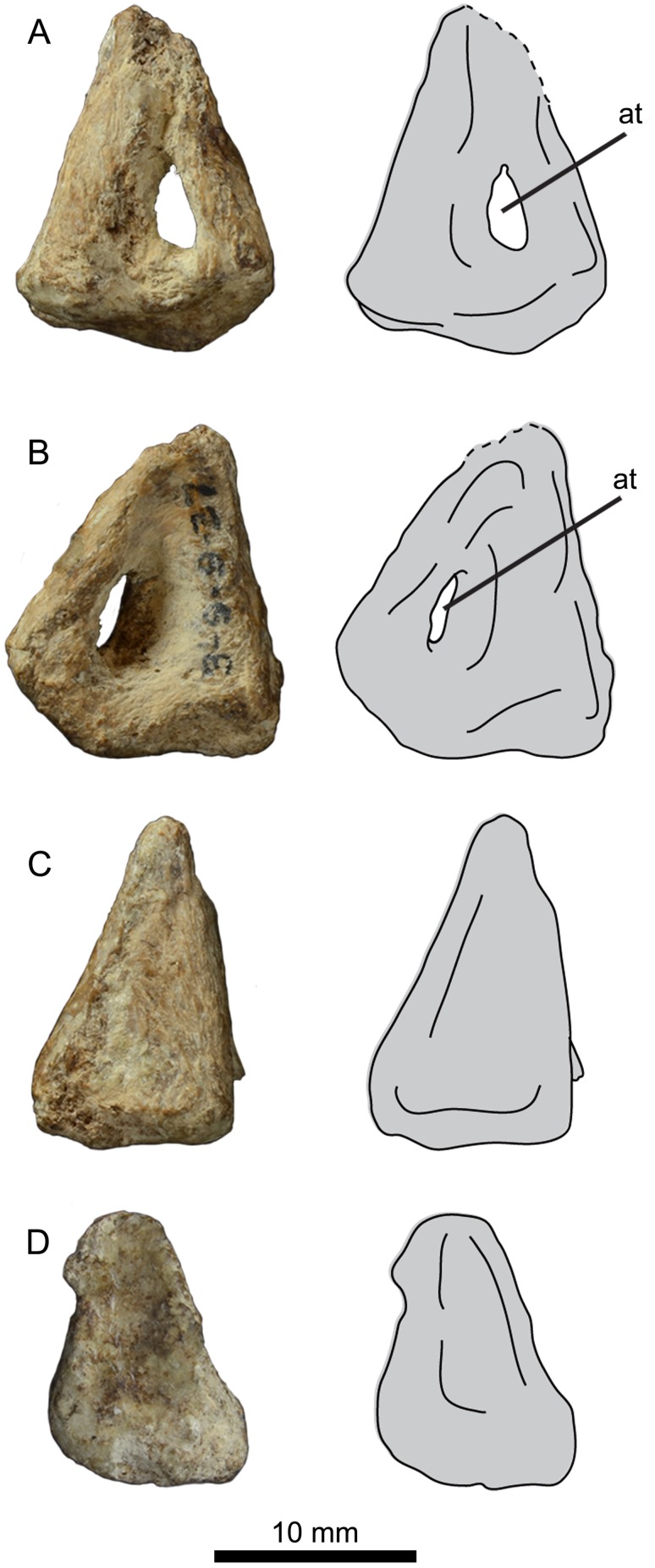
Photograph and interpretative line drawings of the right patella of UNSM 20030 in lateral (A), medial (B), cranial (C), and distal (D) views. Abbreviations: at, foramen for the ambiens tendon.

The right patella is preserved in UNSM 20030 (Fig 14). The patella is small and pyramidal in shape, closely resembling the patella of *B*. *advenus* and unlike the flattened, highly irregular patella of *P*. *alexi* and *H*. *regalis*. The foramen for the ambiens tendon penetrates completely through the bone and is centered over the cranial face. The primary difference between the patella of UNSM 20030 and that of *B*. *advenus* is the flattened caudal face of UNSM 20030, which is curved in *B*. *advenus* and other hesperornithiforms.

### Fibula

**Fig 15:**
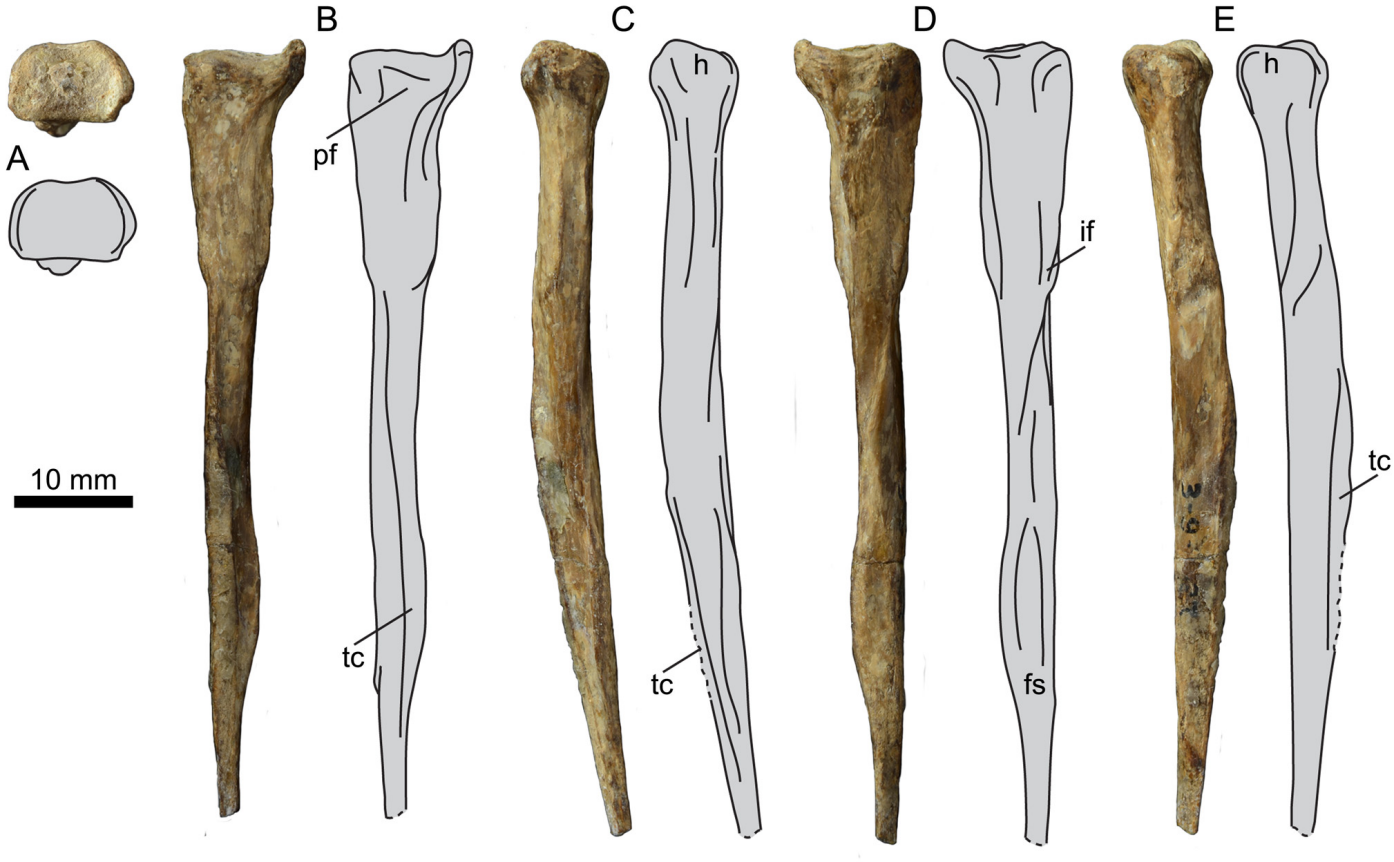
Photographs and interpretative line drawings of the left fibula of UNSM 20030 in proximal (A), lateral (B), cranial (C), medial (D), and caudal (E) views. Abbreviations: fs, fibular spine; h, head; if, tubercle of the *m*. *iliofibuilaris*; pf, popliteal fovea; tc, tibial crest.

UNSM 20030 preserves the proximal end and a portion of the shaft of the left fibula (Fig 15). The fibula is similar to other hesperornithiform fibulae in being crutch-shaped in medial or lateral view, with a caudally expanded proximal end with a u-shaped depression. The degree of excavation of the proximal surface varies widely across hesperornithiforms, however the almost flat surface of UNSM 20030 is unique. To what degree this is a product of erosion of the surface is unclear. The shaft of UNSM 20030 is fairly thick cranio-caudally, narrows abruptly some distance below the proximal surface, and then flares out before narrowing again near the broken distal end.

### Tibiotarsus

**Fig 16:**
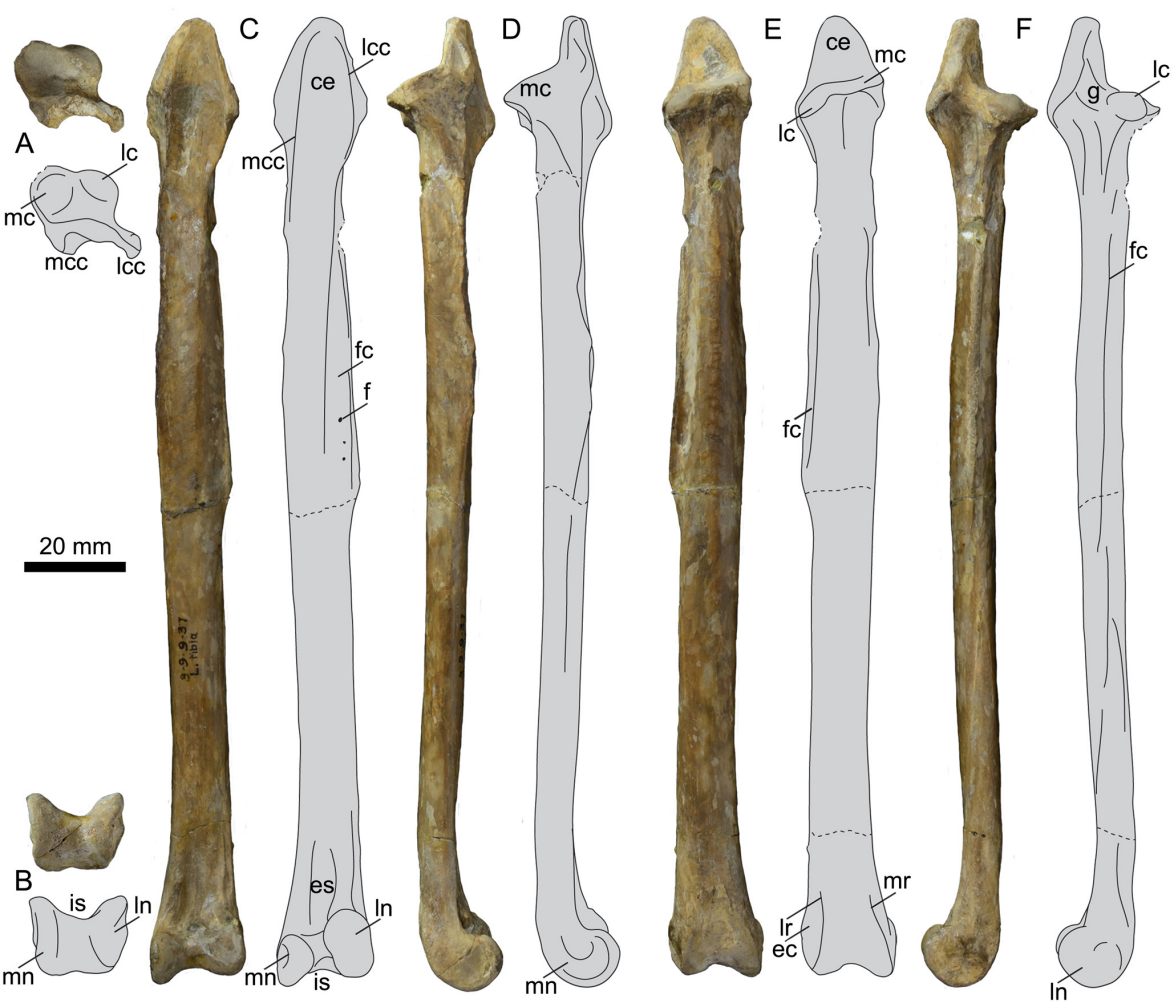
Photographs and interpretative line drawings of the left tibiotarsus of UNSM 20030 in proximal (A), distal (B), cranial (C), medial (D), caudal (E), and lateral (F) views. Abbreviations: ce, cnemial expansion; ec, lateral epicondyle; es, extensor sulcus; f, foramen; fc, fibular crest; is, intercondylar sulcus; it, tibiotarsal incision; lc, lateral cotyla; lcc, lateral cnemial crest; ln, lateral condyle; lr, lateral condylar ridge; mc, medial cotyla; mcc, medial cnemial crest; mn, medial condyle; mr, medial condylar ridge.

**Fig 17:**
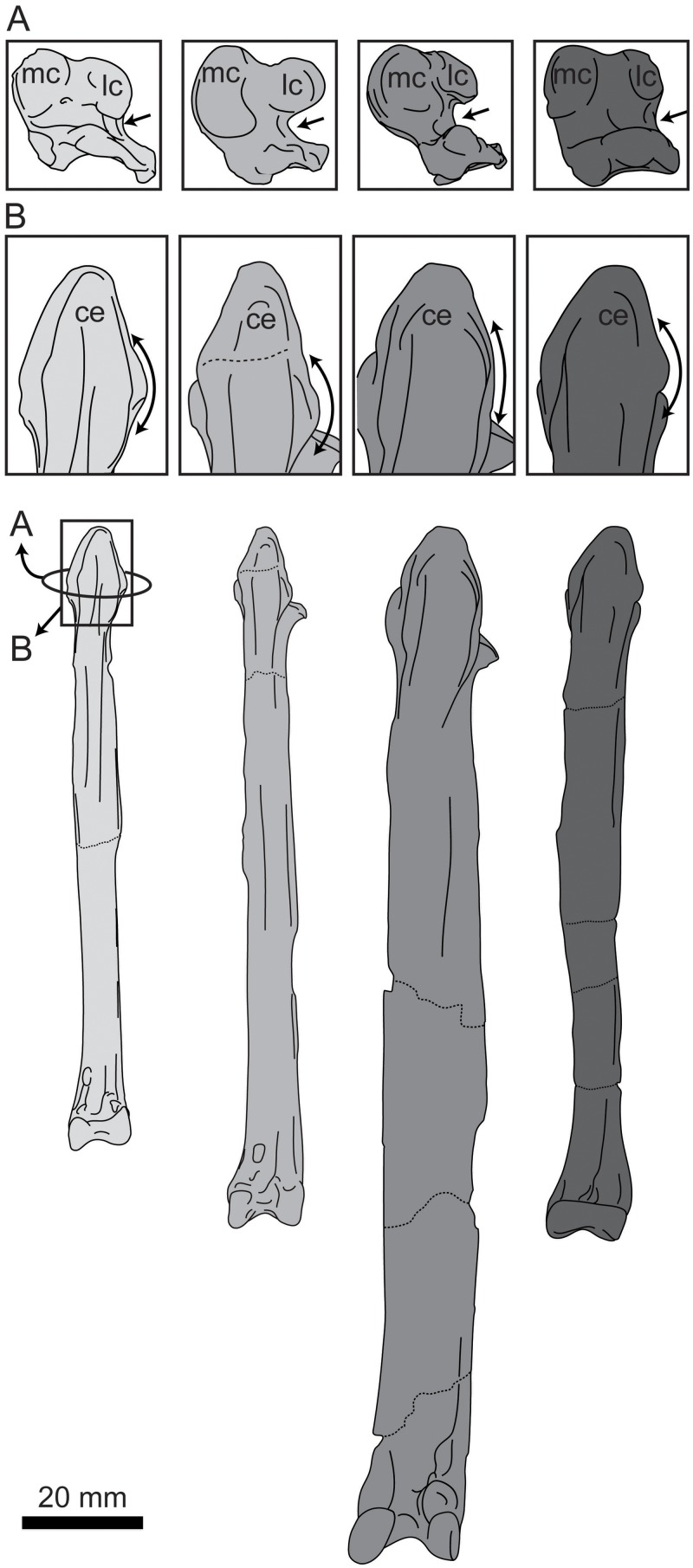
Comparison of the tibiotarsi of hesperornithiforms (left-to-right: *Fumicollis hoffmani* UNSM 20030; *Parahesperornis alexi* KUVP 2287; *Hesperornis regalis* YPM 1200; *Brodavis varneri* SDSM 68430). A, proximal view, arrow indicates the tibial incisure that wraps up from the lateral side onto the proximal surface, cranial to the lateral cotyla. B, cranial view, shape of the distal lateral margin of the cnemial expansion (arrow). Insets are shown scaled to be of similar size to *F*. *hoffmani*. Abbreviations: ce, cnemial expansion; lc, lateral cotyla; mc, medial cotyla.

UNSM 20030 preserves the left and right tibiotarsi (Fig 16), measurements of which are found in Table 3. The left tibiotarsus is completely preserved, although broken cleanly near the midline, while the right preserves only the proximal and distal ends. The proximal tibiotarsus possesses a proximally-projecting triangular expansion formed from the two cnemial crests. The expansion is fairly symmetrical in cranial view, with the medial crest slightly more projected than the lateral crest. Unfortunately the cnemial expansion of *B*. *advenus* is only poorly preserved, making evaluations of its shape difficult. The cnemial expansions of *P*. *alexi* and *H*. *regalis* are fairly symmetrical, however that of *H*. *regalis* is much narrower and shaped more like an oval than a triangle (Fig 17b). In proximal view UNSM 20030 resembles other hesperornithiforms, with the medial cotyla much larger than the lateral and the entire articular surface sloping such that the lateral side is lower than the medial in caudal view. In proximal and lateral views, a deep groove, equivalent to the *incisura tibilais* of Butendiek [13], is present that wraps up onto the proximal surface cranial to the medial cotyla. The incision of UNSM 20030 is intermediate to the faint incision seen in *B*. *advenus* and *Brodavis varneri*, and the more developed incisions of *P*. *alexi* and *H*. *regalis* (Fig 17a). The fibular crest forms from the lateral margin of the lateral cotyla and continues about half-way down the shaft of the tibiotarsus. In *B*. *advenus* the fibular crest does not reach mid-shaft, while in *B*. *varneri*, *P*. *alexi*, and *H*. *regalis* it extends past mid-shaft. The medial cnemial crest continues down the shaft as a thin, sharp ridge, very similar to the state in *P*. *alexi*. The poor preservation of all *B*. *advenus* specimens makes the development of this crest difficult to evaluate, however it is less prominent and shorter in *H*. *regalis*.

**Table 3:** Select measurements of the left tibiotarsus of UNSM 20030, in millimeters.

Length	Length of Fibular Crest	Mid-shaft Diameter	Proximal Medio-Lateral Width	Distal Medio-Lateral Width
190.82	95.01	12.49	17.42	18.87

**Fig 18:**
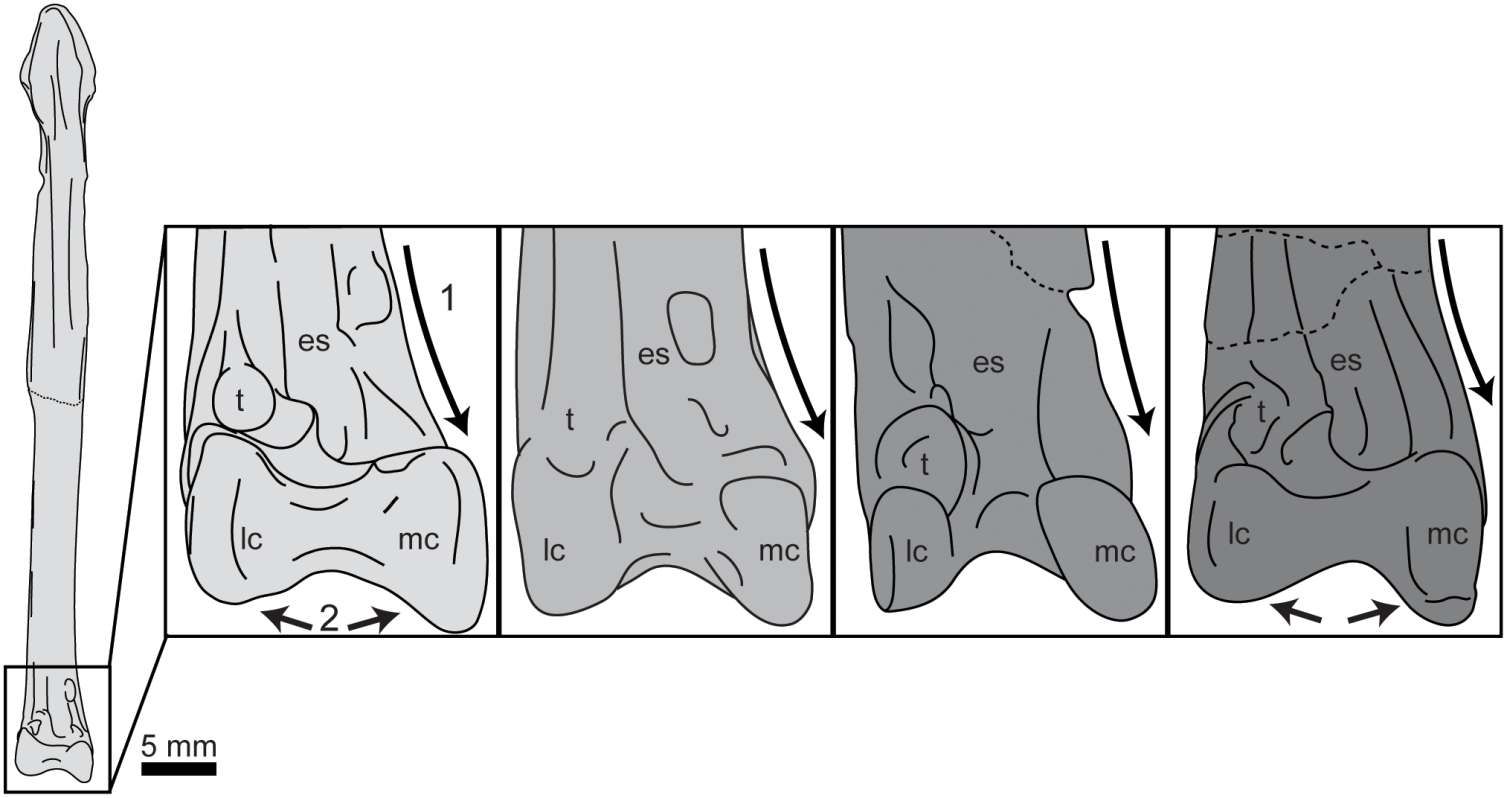
Comparison of the tibiotarsi of hesperornithiforms in cranial view (left-to-right: *Fumicollis hoffmani* UNSM 20030; *Parahesperornis alexi* KUVP 2287; *Hesperornis regalis* YPM 1200; *Baptornis advenus* AMNH 5101). 1, arrow indicates the medial expansion of the distal end, seen to the greatest degree in *F*. *hoffmani* and *P*. *alexi*. 2, arrows indicate the discrepancy in distal extent of the medial and lateral condyles in *F*. *hoffmani* and *B*. *advenus*. Insets are shown scaled to be of similar size to *F*. *hoffmani*. Abbreviations: es, extensor sulcus; lc, lateral condyle; mc, medial condyle; t, *tubercle retinaculi m*. *fibularis*.

**Fig 19:**
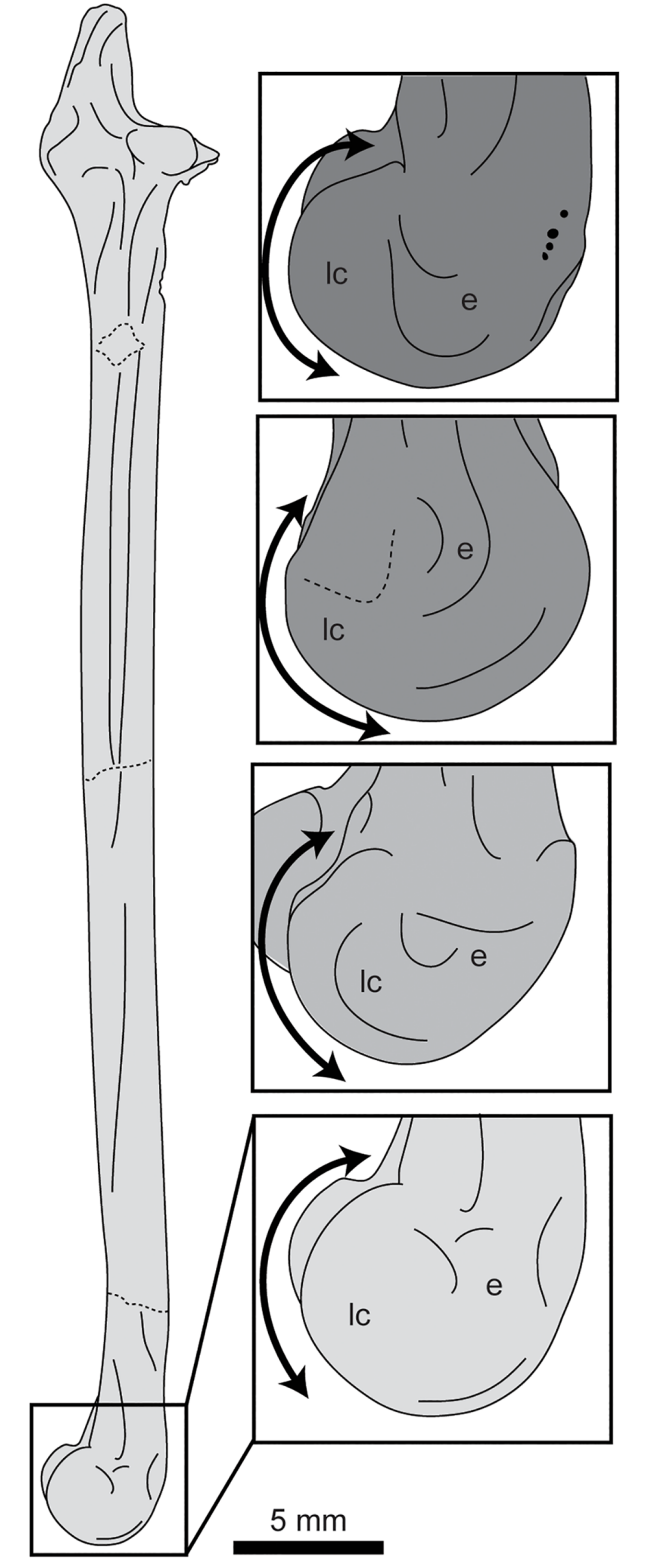
Comparison of the tibiotarsi of hesperornithiforms in lateral view (upper to lower: *Baptornis advenus* AMNH 5101; *Hesperornis regalis* YPM 1200; *Parahesperornis alexi* KUVP 2287; *Fumicollis hoffmani* UNSM 20030). Insets are shown scaled to be of similar size to *F*. *hoffmani*. Arrow indicates the curvature of the cranial edge of the lateral condyle, which varies across the hesperornithiforms shown. Abbreviations: e, epicondylar depression; lc, lateral condyle.

The shaft of the tibiotarsus has an oval cross-section and is fairly straight along its length, with a slight bow in lateral or medial view, as is typical of most hesperornithiforms (except *H*. *regalis*). The shaft does not twist noticeably, as opposed to *H*. *regalis* and *P*. *alexi*, where the proximal end is in cranio-medial view while the distal end is in cranial view. It is not possible to evaluate the degree of shaft twist in *B*. *advenus*, as no specimen is preserved well enough. The shaft expands at the distal end, with the medial side more expanded than the lateral, giving the distal end the appearance of flaring or angling medially in cranial view. This is also observed in *B*. *advenus* and to a greater degree in *H*. *regalis* and *P*. *alexi* (Fig 18a). In cranial view the medial condyle is narrower than the lateral and extends further distally. This is similar to the state in the *B*. *advenus* specimen AMNH 5101, however in the *B*. *advenus* specimen FMNH 395 the condyles are similarly sized with similar distal extents (see [14] for a discussion of the developmental age of FMNH 395). This discrepancy may be explained by the immature age of FMNH 395. In *P*. *alexi* and *H*. *regalis* the medial condyle is narrower than the lateral in cranial view, however both have a similar distal extent (Fig 18b). In medial view the medial condyle is hook-shaped and highly rounded, extending cranially. *B*. *advenus* lacks the hook-shaped aspect to the medial condyle and is instead more circular in medial view. In *P*. *alexi* and *H*. *regalis* the medial condyle is very narrow cranio-caudally and nearly circular, shifted disto-cranially from the shaft, as opposed to primarily cranially in *B*. *advenus* and UNSM 20030. In caudal view the condyles extend onto the shaft as thin ridges angled toward the midline of the shaft, as in other hesperornithiforms. In lateral view the lateral condyle of UNSM 20030 is rounded, extending cranially from the shaft, and appears similar to that of *P*. *alexi*. This extension is much less in UNSM 20030 than in *B*. *advenus*, where the lateral condyle is almost j-shaped in lateral view. *H*. *regalis* has a semi-circular lateral condyle centered below the shaft in lateral view (Fig 19). The intercondylar sulcus of UNSM 20030 is broad and rounded in cranial view, more so than in other hesperornithiforms. The ascending process of the astragalus is fully fused to the distal lateral shaft of the tibiotarsus, supporting a mature age for the individual. In caudal view the intercondylar sulcus is similar to that of other hesperornithiforms, being slightly asymmetrical, with the medial side more steeply angled than the lateral. In distal view, the intercondylar sulcus appears to be shallower than in other hesperornithiforms. The cranio-caudal lengths of the medial and lateral condyles are nearly equal, as in *B*. *advenus* and in contrast with *P*. *alexi* and *H*. *regalis*, where the medial condyle is much shorter than the lateral.

### Tarsometatarsus

**Fig 20:**
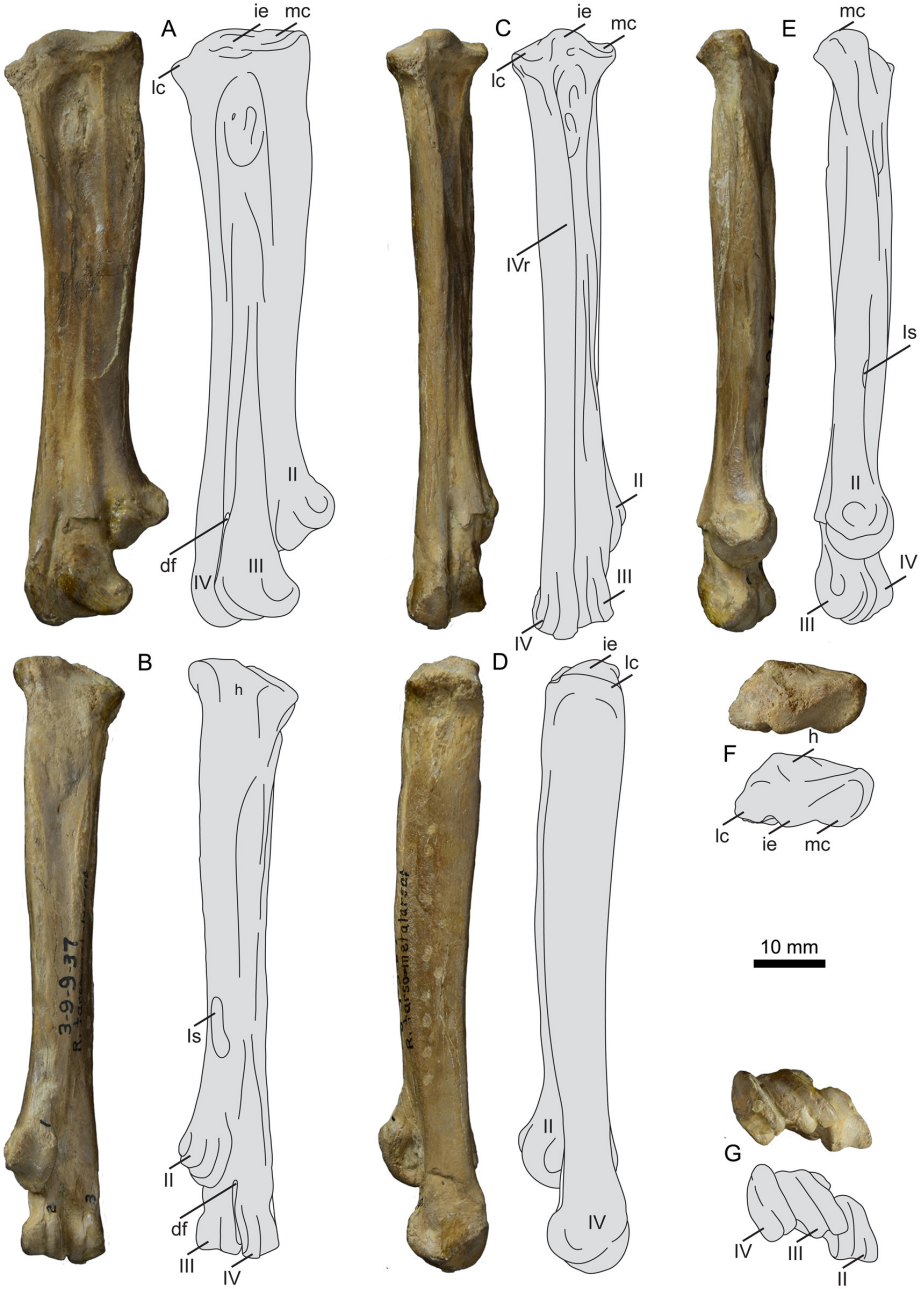
Photographs and interpretative line drawings of the right tarsometatarsus of UNSM 20030 in dorsal (A, C), plantar (B), lateral (D), medial (E), proximal (F), and distal (G) views. Abbreviations: df, distal foramen; ie, intercotylar eminence; h, rudimentary hypotarsus; lc, lateral cotyla; mc, medial cotyla; II–IV, trochlea of metatarsals II–IV; Is, scar for metatarsal I; IVr, ridge formed from metatarsal IV.

UNSM 20030 preserves the complete left tarsometatarsus (Fig 20), measurements of which are found in Table 4. Metatarsals II-IV are completely fused along their length with the individual metatarsals separated by slight grooves along the length of the element. The tarsometatarsus of UNSM 20030 displays a stacking or shingling of the metatarsals, such that the trochlea of metatarsal IV extends further dorsally than that of III, which is in turn more dorsal than trochlea II. This is also seen in *P*. *alexi* and *H*. *regalis*. This shingling is only minimally observed in *B*. *advenus* or *Brodavis*, where II and IV are similar in dorsal extent while II is offset plantarly. Like other hesperornithiforms, the shaft of UNSM 20030 is twisted, such that when the proximal end of the element is in dorsal view, the distal end is in medio-dorsal view. Within hesperornithiforms, the shaft of the tarsometatarsus of *B*. *advenus* and *B*. *varneri* is only slightly twisted, while that of UNSM 20030, *P*. *alexi*, and *H*. *regalis* is more dramatically twisted. This twist combined with the shingling of the metatarsals gives the tarsometatarsus a very narrow, straight appearance when the distal end is in dorsal view (Fig 20c) and a much broader, curvy appearance when the proximal end is in dorsal view (Fig 20a).

**Table 4:** Select measurements of the right tarsometatarsus of UNSM 20030, in millimeters.

Length	Medio-lateral Mid-shaft Width	Proximal Depth	Proximal Width	Distal Width	Medio-lateral Width of Trochlea III	Medio-lateral Width of Trochlea IV	Distal Displacement of Scar for Metatarsal I
83.72	9.74	10.72	17.45	11.63	5.12	5.13	46.52

**Fig 21:**
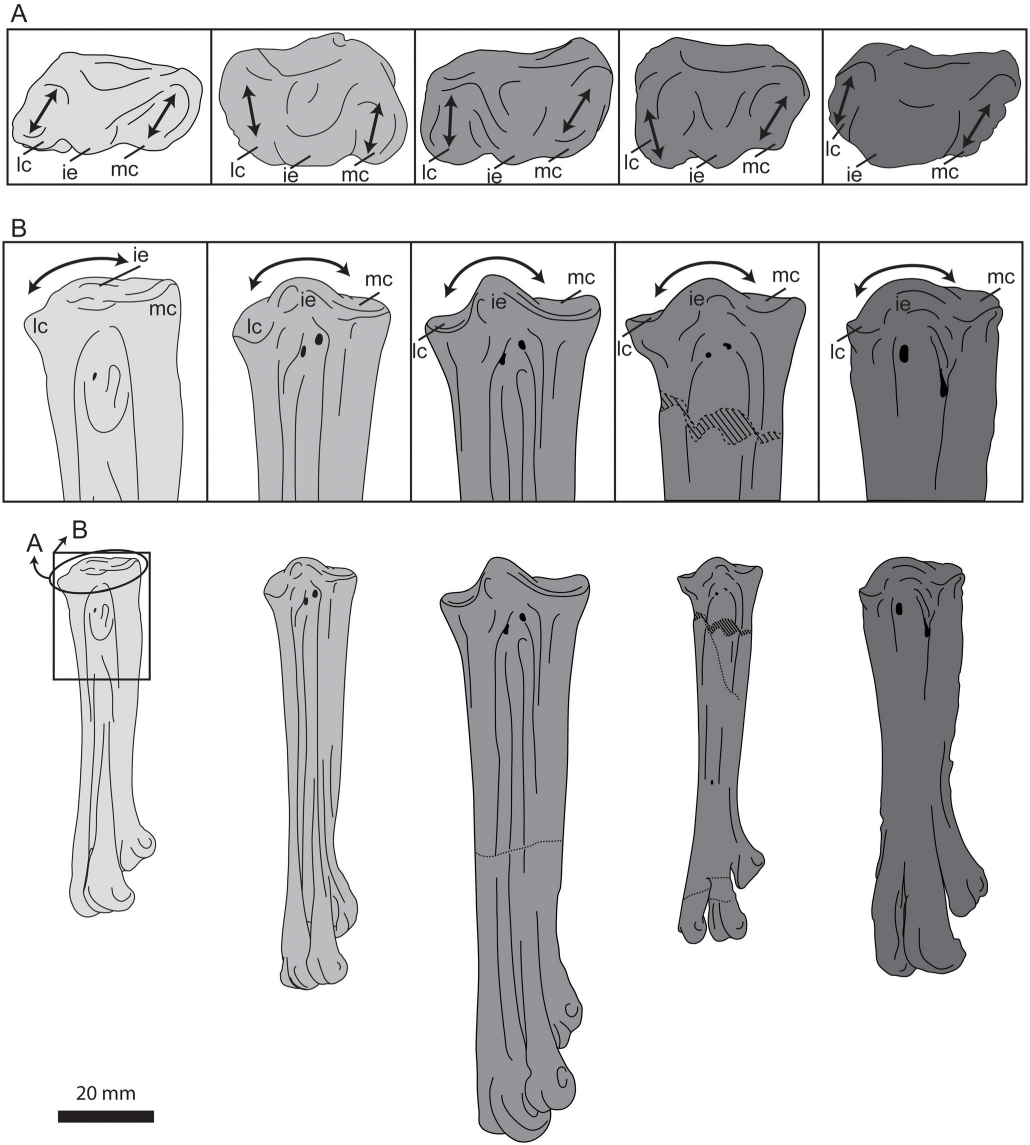
Comparison of the tarsometatarsi of hesperornithiforms in dorsal view (left-to-right: *Fumicollis hoffmani* UNSM 20030; *Parahesperornis alexi* KUVP 2287; *Hesperornis regalis* YPM 1200; *Baptornis advenus* AMNH 5101; *Brodavis varneri* SDSM 68430). A, proximal view, shape of the proximal articular surface, with arrows indicating the widths and orientations of the medial and lateral cotylae. B, dorsal view, shape and orientation of the intercotylar eminence (arrow). Insets are shown scaled to be of similar size to *F*. *hoffmani*. Abbreviations: ie, intercotylar eminence; lc, lateral cotyla; mc, medial cotyla.

**Fig 22:**
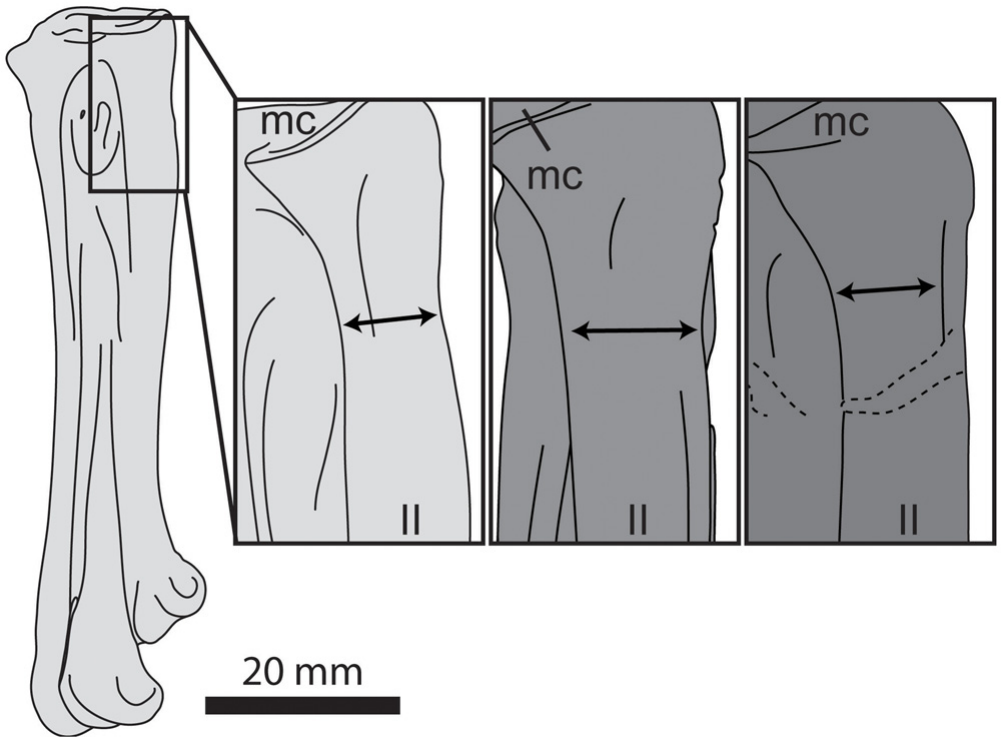
Comparison of the tarsometatarsi of hesperornithiforms in dorsal view (left-to-right: *Fumicollis hoffmani* UNSM 20030; *Hesperornis regalis* YPM 1200; *Baptornis advenus* AMNH 5101). Arrow indicates the proximal narrowing of the neck of metatarsal II, which is most pronounced in *F*. *hoffmani*. Insets are shown scaled to be of similar size to *F*. *hoffmani*. Abbreviations: II, trochlea of metatarsal II; mc, medial cotyla.

In proximal view the articular surface of the tarsometatarsus is very narrow and rectangular in outline, much more so than in other hesperornithiforms, which tend to be boxier in proximal outline. The medial and lateral cotylae are similarly sized in UNSM 20030, which is more similar to the morphology of *P*. *alexi* and *H*. *regalis* and different from that of *B*. *advenus* and *Brodavis varneri*, where the lateral cotyla is much longer dorso-plantarly than the medial (Fig 21a). As in other hesperornithiforms, the intercotylar eminence is offset dorsally, such that the dorsal margin of the articular surface is shaped like an inverted ‘W’ in proximal view. Also like other hesperornithiforms, the proximal articular surface forms an angle with the longitudinal axis of the shaft in dorsal or plantar view, with the medial cotyla higher than the lateral. The intercotylar eminence is small and slightly rounded, most similar to the case in *B*. *varneri*, while *B*. *advenus*, *P*. *alexi*, and *H*. *regalis* the intercotylar eminence is progressively higher and more peaked (Fig 21b). As in all hesperornithiforms, the intercotylar eminence is angled laterally in dorsal view. Metatarsals II and IV form sharp ridges immediately below the proximal articular surface in dorsal view, with metatarsal III recessed between II and IV. The proximal dorsal face of metatarsal III is rugose and has two proximal foramina that do not appear to penetrate to the plantar side of the element. In medial view the proximal end of metatarsal II is sloped downward at the dorsal tip and bears a broad, shallow depression not seen in other hesperornithiforms. The shaft of metatarsal II narrows abruptly into a neck just below the proximal surface, similar to the state in *P*. *alexi* or *H*. *regalis* and more exaggerated than *B*. *advenus* (Fig 22). In plantar view the proximal end of the tarsometatarsus bears a slightly rugose, raised area in place of a fully developed hypotarsus, as seen in other hesperornithiforms. In lateral view the proximal end of metatarsal IV is rounded and bears a deep depression, as in other hesperornithiforms.

**Fig 23:**
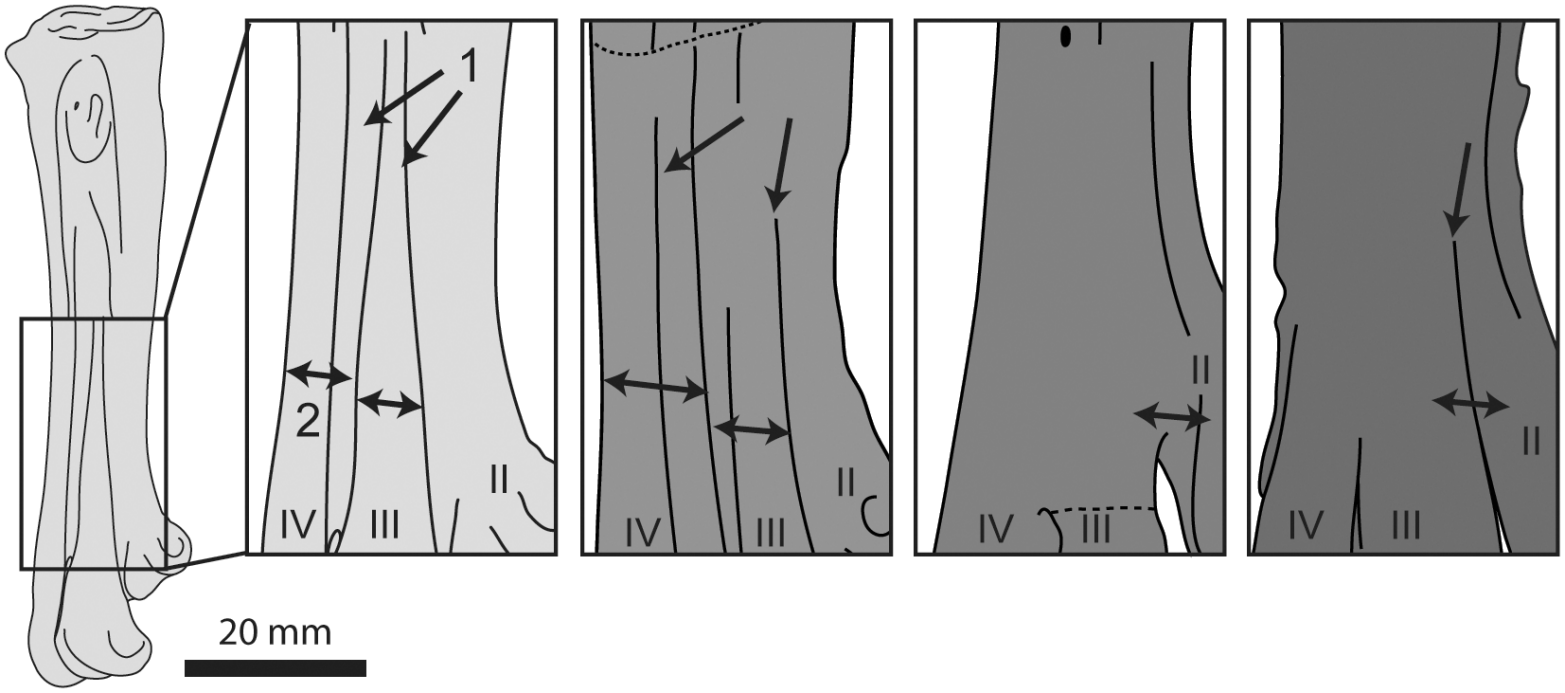
Comparison of the tarsometatarsi of hesperornithiforms in dorsal view (left-to-right: *Fumicollis hoffmani* UNSM 20030; *Hesperornis regalis* YPM 1200; *Baptornis advenus* AMNH 5101; *Brodavis varneri* SDSM 68430). 1, arrows indicate grooves separating the metatarsals along the shaft of the tarsometatarsus; 2, arrows indicate the shingled arrangement of the metatarsals. Insets are shown scaled to be of similar size to *F*. *hoffmani*. Abbreviations: II–IV, trochlea of metatarsals II–IV.

The ridge formed by metatarsal IV continues down the length of the shaft of the tarsometatarsus, terminating at trochlea IV. This is also seen in *P*. *alexi* and *H*. *regalis*, while in *B*. *advenus* and *B*. *varneri* the ridge flattens out near midshaft (Fig 23). The grooves separating the metatarsals of UNSM 20030 are better developed than in *B*. *advenus* but not as deeply excavated as in *P*. *alexi* and *H*. *regalis*. The plantar surface of the shaft is fairly flat with sharply defined medial and lateral margins that taper continuously to the distal end, as in other hesperornithiforms. The midline of the plantar surface of the shaft is slightly depressed along its length, like in *P*. *alexi*, *H*. *regalis*, and *B*. *varneri* and unlike the featureless surface in *B*. *advenus*. In medial or lateral view the shaft of the tarsometatarsus of UNSM 20030 is bowed. This is also seen in *B*. *advenus* but not in *P*. *alexi* and *H*. *regalis*.

**Fig 24:**
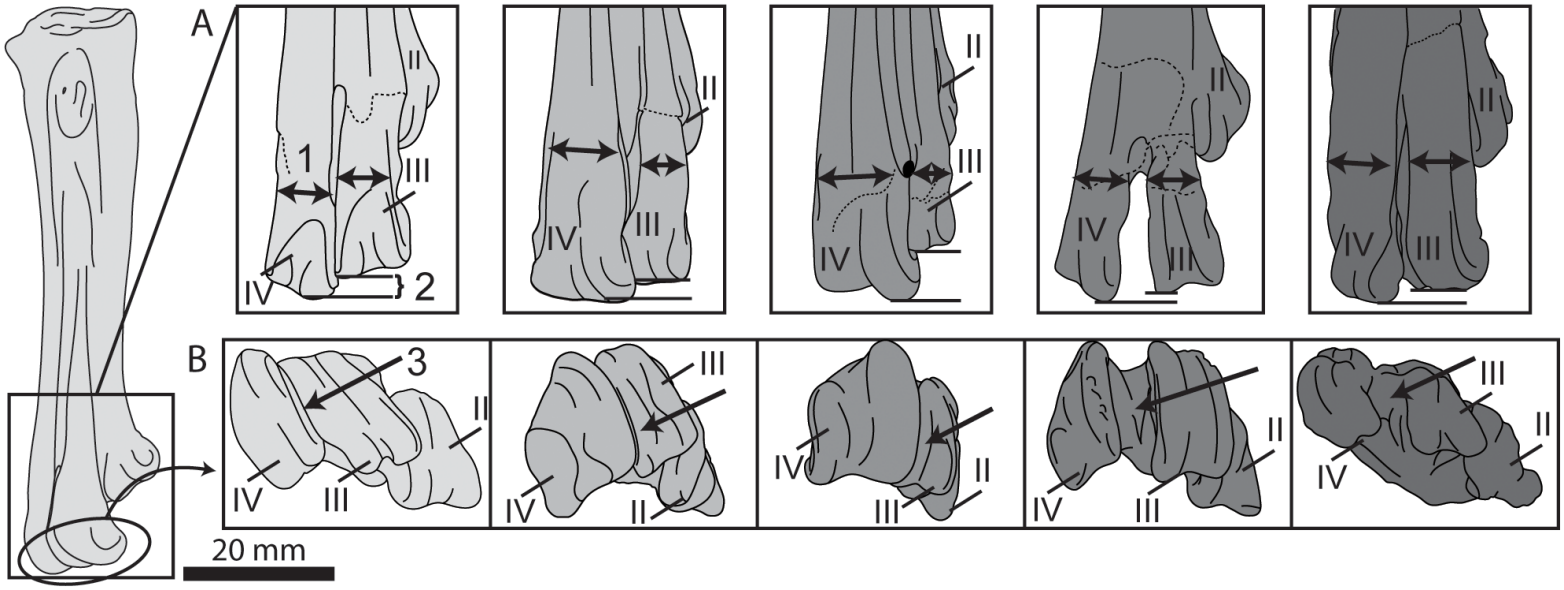
Comparison of the tarsometatarsi of hesperornithiforms (left-to-right: *Fumicollis hoffmani* UNSM 20030; *Parahesperornis alexi* KUVP 2287; *Hesperornis regalis* YPM 1200; *Baptornis advenu*s AMNH 5101; *Brodavis varneri* SDSM 68430). A, dorsal view, relative widths (arrows, 1) and distal extents (2) of metatarsals III and IV. B, distal view, arrangement of the metatarsal trochleae, with arrow (3) indicating spacing between the trochleae of metatarsals III and IV. Insets are shown scaled to be of similar size to *F*. *hoffmani*. Abbreviations: II–IV, trochlea of metatarsals II–IV.

Like all hesperornithiforms, the trochlea of metatarsal II is wedge-shaped and more narrow medio-laterally than trochlea III or IV in dorsal view, and is shifted plantarly behind the trochlea of metatarsal III. In *B*. *advenus* the trochleae are fairly broadly spaced, with a distinct intercondylar space between each. However, in UNSM 20030, *P*. *alexi*, and *H*. *regalis* this space is reduced to near absence, with trochlea III and IV touching along their length and trochlea II only very slightly spaced from trochlea III (Fig 24a). The dorsal widths of trochlea III and IV are similar, as in *B*. *advenus* and unlike *P*. *alexi* and *H*. *regalis*, where trochlea IV is significantly wider than III (Fig 24b). In plantar view, trochlea III and IV extend distally a similar amount, like in *B*. *advenus* and *P*. *alexi* and different from *H*. *regalis*, where trochlea IV extends much further distally than III. Like *P*. *alexi* and *H*. *regalis*, the medial ridge of trochlea IV is greatly enlarged as compared to the lateral.

### Pedal phalanges

**Fig 25:**
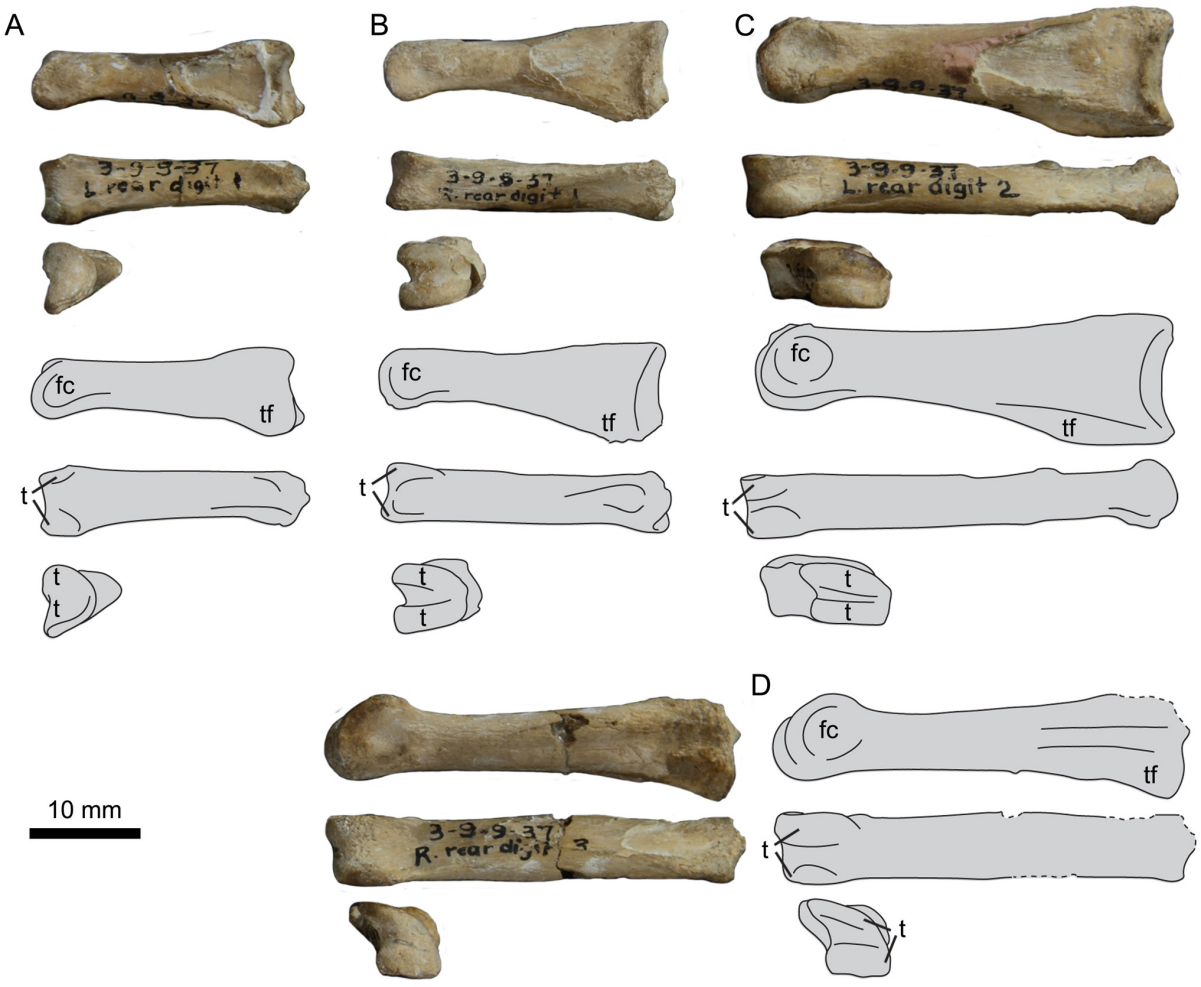
Photographs and interpretative line drawings of the pedal phalanges of *Fumicollis hoffmani*. Phalanx III:3 (A), III:2 (B), III:1 (C), and II:1 (D) in (top to bottom): lateral, dorsal, and proximal views. Abbreviations: fc, fovea for the collateral ligament; t, articular trochlea; tf, flexor tubercle.

UNSM 20030 preserves the non-ungual phalanges from the left third toe (III:1, III:2, III:3), the two proximal-most phalanges from the right third toe (III:1, III:2), and the proximal-most phalanx from the left second toe (II:1) (Fig 25), measurements of which are found in Table 5. As a result of the unique trochlear morphology of the tarsometatarsus, the proximal phalanges of hesperornithiforms are fairly distinctive. Phalanx III:1 is very narrow medio-laterally with a triangular shape in medial or lateral view, with a straight dorsal margin and a highly curved plantar margin. This is most easily seen in *H*. *regalis*, while UNSM 20030 is less exaggerated than either *P*. *alexi* or *H*. *regalis*. Phalanx III:1 is not known for *B*. *advenus*, but comparison to III:2 and III:3 indicate that it was likely even less exaggerated in shape than UNSM 20030.

**Table 5:** Select measurements of the pedal phalanges of UNSM 20030, in millimeters.

	Length	Mid-shaft Diameter
Phalanx II:1	37.42	5.21
Phalanx III:1	37.62	6.54
Phalanx III:2	25.83	4.74
Phalanx III:3	24.43	4.48

### Thoracic ribs

**Fig 26:**
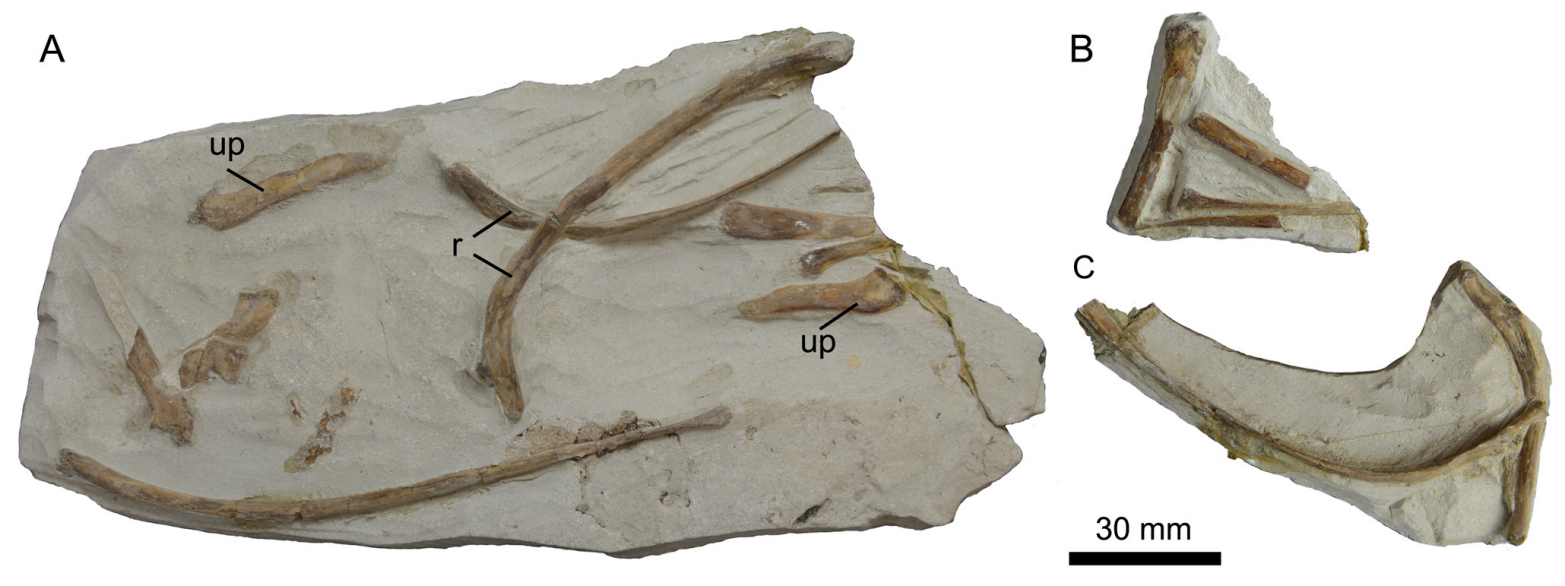
Three slabs (A-C) preserving thoracic ribs and uncinate processes of *Fumicollis hoffmani*. Abbreviations: r, ribs; up, uncinated process.

Fragments of a number of thoracic ribs are preserved on three separate slabs (Fig 26). While most are poorly preserved, one rib head is visible in lateral view. The head is crutch-shaped, with a robust flange extending proximal to the articular surface. The uncinate processes appear to be unfused from the ribs, as in other hesperornithiforms.

## Discussion

*Fumicollis hoffmani*, UNSM 20030 is notable in that it combines a suite of basal traits commonly seen in smaller hesperornithiforms such as *Baptornis advenus* (i.e. proximal and distal ends of femur not expanded, elongate pre-acetabular ilium, small and pyramidal patella) with derived characters common to larger hesperornithiforms such as *Hesperornis regalis* (i.e. dorsal ridge on trochlea of metatarsal IV, plantarly-projected curve in the distal shaft of phalanx III:1). In this sense, UNSM 20030 is similar to *Parahesperornis alexi*, which has also been identified as phylogenetically intermediate to *B*. *advenus* and *H*. *regalis* [9, 15]. UNSM 20030 and the holotype of *P*. *alexi* (KUVP 2287) are similarly sized, particularly as compared to the much larger specimens of *H*. *regalis*, and they appear to be fully mature. In particular, the components of the tarsometatarsus and tibiotarsus are fully fused, with suture lines not visible between the metatarsals or around the ascending process of the astragalus. Additional indicators of a mature age include the complete fusion of the acetabulum and the absence of neurocentral surtures in the vertebrae. Despite the size differences, in terms of morphology *P*. *alexi* is much more similar to *H*. *regalis* than to *Fumicollis hoffmani*, which bears a number of anatomical traits that are similar to those of *B*. *advenus* (particularly in the femur). This combination of features is all the more striking given the similar age and geographic overlap of these taxa. *B*. *advenus*, *H*. *regalis*, *P*. *alexi*, and *F*. *hoffmani* are all known from the upper portion of the Late Cretaceous Smoky Hill Member of the Niobrara Chalk in Logan County (Kansas), strata that only spans around five million years of depositional history [16], yet contains the highest known taxonomic diversity of hesperornithiforms (Fig 2). The co-existence in geologic, if not also ecologic, time of numerous diving birds with a wide range of body sizes and specializations suggests a high degree of niche partitioning [7, 8, 17].

This possible overlap in time and space of birds similarly adapted to a highly specialized life style is echoed today in the distribution of penguins in the peninsular region of Antarctica, including Alexander, Orkney, and Shetland Islands. In this region up to six different penguin species are found, ranging in size from 4 kilograms (*Pygoscelis antarctica*) to around 30 kilograms (*Aptenodytes forsteri*). Research has determined that these birds avoid competition in a variety of ways: reliance on different food types [18]; use of different foraging locations or water depths [19, 20]; and use of different foraging times (diurnal vs. nocturnal [19]). While the co-existence of all of the Smoky Hill hesperornithiforms in ecologic time may never be determined, their proximity in geologic time suggests the possibility of a similarly complex ecosystem to that in which Antarctica’s penguins coexist today. Therefore, one or all of these niche partitioning methods may have been adopted by the hesperornithiforms of the Western Interior Seaway in order for this high diversity of divers to coexist.

The relative abilities of different hesperornithiforms to access waters at greater depths or further distances from shore would have contributed to niche partitioning. While *H*. *regalis* and *P*. *alexi* have numerous skeletal features indicative of advanced diving specializations [8, 12], many of these are absent in *F*. *hoffmani*, thus suggesting lesser diving capabilities. For example, the comparatively reduced femoral trochanter and patella of UNSM 20030 would limit the area available for the attachment of several muscles (i.e., the lateral and medial obturatorius, medial and cranial iliotrochantericus, medial femorotibialis, and medial gastrocnemius) crucial to orienting the hindlimb for the power stroke and providing propulsive force during diving [12], thus possibly limiting the speed of *Fumicollis hoffmani*. The highly specialized peg-and-socket articulation of the fourth toe in *H*. *regalis* is absent in UNSM 20030, suggesting that the toes of *F*. *hoffmani* could not be folded as efficiently during the return stroke, thus increasing drag and reducing swimming speed. Furthermore, the small body size of *F*. *hoffmani* would have limited its depth range when diving, as documented by numerous studies of modern diving animals that demonstrate a positive correlation between body size and diving depth [21–23]. However, *F*. *hoffmani* does preserve diving adaptations more advanced than those of the coexisting *B*. *advenus*, such as the shingled arrangement of the metatarsals. This stacking arrangement has been shown to assist grebes in folding their toes together during the return stroke while swimming, thus reducing drag [24]. Therefore, the available morphological evidence indicates that *F*. *hoffmani* was a more advanced diver than *B*. *advenus*, perhaps allowing access to deeper waters for hunting, however not as advanced as *H*. *regalis* and *P*. *alexi*.

## Conclusions

UNSM 20030 provides us with a novel hesperornithiform, *Fumicollis hoffmani*, that is morphologically transitional between the smaller, more primitive *Baptornis advenus* and the larger, more derived *Hesperornis regalis* and *Parahesperornis alexi*. Phylogenetic analysis of *F*. *hoffmani* and other hesperornithiforms has supported an intermediate placement of *F*. *hoffmani* between *B*. *advenus* and *H*. *regalis* and *P*. *alexi* [8], highlighting the significance of this well-preserved specimen in furthering our understanding of the evolution of diving specializations in a group of Late Cretaceous birds. The identification of *Fumicollis hoffmani*, a new taxon displaying a complex mosaic of anatomical traits present in other hesperornithiforms, further illustrates the taxonomic diversity reached by these Late Cretaceous birds. Given the overlap in geologic time and space among some hesperornithiforms, including *Fumicollis hoffmani*, physical distinctions may have played a role in niche partitioning within the Late Cretaceous diving birds of the Western Interior Seaway of Kansas.

## Supporting Information

S1 Appendix Character state disagreement between UNSM 20030 and *Baptornis*. Characters from the analysis of Bell and Chiappe [8] for which UNSM 20030 was coded differently than other specimens of *Baptornis*.
(DOCX)

## Abbreviations

AMNH: American Museum of Natural History
FMNH: Field Museum of Natural History
KUVP: University of Kansas Museum of Paleontology
NMNH: National Museum of Natural History, Smithsonian Institute
SDSM: South Dakota School of Mines
UNSM: University of Nebraska State Museum
YPM: Yale Peabody Museum
