# Supplementary material for: Identification of a New Hesperornithiform from the Cretaceous Niobrara Chalk and Implications for Ecologic Diversity among Early Diving Birds
Source: PLoS One. 2015 Nov 18;10(11):e0141690. doi: 10.1371/journal.pone.0141690 (PMC4651437; doi:10.1371/journal.pone.0141690)
Supplement: S1 Appendix — Characters from the analysis of Bell and Chiappe [8] for which UNSM 20030 was coded differently than other specimens of Baptornis. (DOCX) [file pone.0141690.s001.docx]

**Appendix I**

**Character state disagreement**

The following characters from the analysis of Bell and Chiappe [8] had conflicting codings for UNSM 20030 and specimens assigned to *Baptornis advenus.* Character numbers reflect those used in the original analysis [8]. *Baptornis* specimens coded were: YPM 1476 (*B. advenus* holotyope); FHSM 6318; KUVP 2290; AMNH 5101.

| Character number | Character | UNSM 20030 *Fumicollis hoffmani* | YPM 1476 *Baptornis advenus* | other *Baptornis* specimens |
| --- | --- | --- | --- | --- |
| 117 | Femur in caudal view, medial margin of shaft distal to head: concave, shallow excavation (0); nearly straight (1); concave, deepest excavation offset proximally (2), slight s-shape (3), dramatic s-shape, proximal half appears waisted (4). | 2 | ? | 0 |
| 120 | Femur shaft: cranially convex (in medial view, proximal and distal ends inflected caudally): absent (0); slight inflection (1), dramatic curvature (2). | 2 | ? | 0 |
| 121 | Femur, trochanter and head: nearly continuous or separated by a shallow notch (0); separated by a deep notch (1). | 1 | ? | 0 |
| 128 | Femur, cranial view, trochanter extends proximally: similar extent (0); further (1); less far (2) than head. **when femur aligned along axis between notch between head and trochanter and intercondylar sulcus* | 1 | ? | 0 |
| 135 | Femur, medial condyle in caudal view: sub-circular or oval (0); kidney bean shaped (1). | 1 | ? | 0 |
| 141 | Femur, lateral view, lateral condyle: merges smoothly into shaft (0); constricts into neck before widening at shaft (1). | 1 | ? | 0 |
| 142 | Femur, distal surface of lateral condyle: featureless curve (0); small bump or rounded prominence (1); projected, flattened prominence (2). *Ordered* | 2 | ? | 1 |
| 143 | Femur, caudal view, fibular condyle bears a small depression on distal-most portion: absent (0); present (1). | 1 | ? | 0 |
| 159 | Tibiotarsus, medial view, shaft: is fairly straight (0), is bowed (1). | 1 | ? | 0 |
| 160 | Tibiotarsus, fibular crest extends approximately: over half-way (0); half-way (1) down shaft; restricted to upper 1/3 of shaft (2). | 0 | ? | 1 |
| 169 | Tibiotarsus, cranial view, medial condyle: similar length to (0); shorter than (1) lateral condyle. | 1 | ? | 0 |
| 174 | Fibula, cranial margin as it approaches proximal end: continues in a smooth curve, widening slightly (0); bulges cranially (1). | 0 | ? | 1 |
| 177 | Tarsometatarsus, intertrochlear space between trochlea of metatarsals III and IV: widely spaced (0); reduced, narrow (1); absent, trochlea III and IV touch (2). | 2 | 1 | 0/1 |
| 186 | Tarsometatarsus, medial and plantar views: proximal plantar margin of metatarsal II possesses a bulbous flange: absent (0); slight (1); enlarged or bulbous (2). *Ordered* | 1 | ? | 0 |
| 188 | Tarsometatarsus, lateral view, round depression on proximal-most face of metatarsal IV: weakly developed (0); well-developed (1). | 1 | ? | 0 |
| 191 | Tarsometatarsus, dorsal view, relative position of metatarsals at midshaft: aligned (0); IV and III aligned, II shifted plantarly (1); IV, III, and II progressively displaced plantarly (stacked or shingled appearance) (2). *Ordered* | 2 | ? | 1 |
| 193 | Tarsometatarsus, shaft twisted laterally - when the distal end is in dorsal view, the proximal end is in: dorsal view (no or minimal twisting) (0); dorso-lateral view (1). | 1 | ? | 0 |
| 194 | Tarsometatarsus, dorsal view, grooves separating metatarsals: absent - metatarsals unfused along shaft (0); absent - seam or crack only (1); prominent - deep groove between metatarsals III and IV along entire length of shaft (2). | 2 | 1 | 1 |
| 195 | Tarsometatarsus, ridge on the dorsal surface of metatarsal IV: absent (0); extends to midshaft (1); extends to trochlea (2). | 2 | ? | 1 |
| 196 | Tarsometatarsus, lateral view, metatarsal IV shaft: tapers evenly to distal end (0); widest at midshaft, tapers at both proximal and distal ends (1). | 1 | ? | 0 |
| 200 | Tarsometatarsus, plantar view, intertrochlear incision between III and IV: absent, metatarsals unfused (0); wedge-shaped (1); enclosed oval or tear-drop shape with rounded proximal end (2); round (3). | 2 | 1 | 1 |
| 201 | Tarsometatarsus, distal extent of trochlea IV: not as far as (0); to a similar level as (1); slightly further than (2); or markedly further than (3) trochlea III. *Ordered* | 2 | 1 | 1 |
